# A Non-Targeted Approach Unravels the Volatile Network in Peach Fruit

**DOI:** 10.1371/journal.pone.0038992

**Published:** 2012-06-22

**Authors:** Gerardo Sánchez, Cristina Besada, María Luisa Badenes, Antonio José Monforte, Antonio Granell

**Affiliations:** 1 Instituto de Biología Molecular y Celular de Plantas (IBMCP, CSIC-UPV), Valencia, Spain; 2 Instituto Nacional de Tecnología Agropecuaria (INTA), Buenos Aires, Argentine; 3 Instituto Valenciano de Investigaciones Agrarias (IVIA), Valencia, Spain; University of Nottingham, United Kingdom

## Abstract

Volatile compounds represent an important part of the plant metabolome and are of particular agronomic and biological interest due to their contribution to fruit aroma and flavor and therefore to fruit quality. By using a non-targeted approach based on HS-SPME-GC-MS, the volatile-compound complement of peach fruit was described. A total of 110 volatile compounds (including alcohols, ketones, aldehydes, esters, lactones, carboxylic acids, phenolics and terpenoids) were identified and quantified in peach fruit samples from different genetic backgrounds, locations, maturity stages and physiological responses. By using a combination of hierarchical cluster analysis and metabolomic correlation network analysis we found that previously known peach fruit volatiles are clustered according to their chemical nature or known biosynthetic pathways. Moreover, novel volatiles that had not yet been described in peach were identified and assigned to co-regulated groups. In addition, our analyses showed that most of the co-regulated groups showed good intergroup correlations that are therefore consistent with the existence of a higher level of regulation orchestrating volatile production under different conditions and/or developmental stages. In addition, this volatile network of interactions provides the ground information for future biochemical studies as well as a useful route map for breeding or biotechnological purposes.

## Introduction

Peach (*Prunus persica* L.) is an economically important crop with an expanding world production situated at 20 million tons in 2010 [1]. Nevertheless, peach consumption (2 kg of fruit per capita per year) is still considered low when compared to other fresh fruit such as apple (16 kg) or banana (9 kg) [2]. One straightforward way to enhance peach consumption would appear to be the improvement of fruit quality, as consumers have been complaining about the quality of peaches since the early 90 s [3]. Aroma, along with fruit firmness and colour, are the most important factors that contribute to peach quality according to consumers [4]. Volatile organic compounds (VOCs) define fruit aroma and, in combination with sugars and organic acids, contribute to the overall peach flavor. Peach volatiles have been studied intensively, and around 100 volatiles, including alcohols, aldehydes, esters, terpenoids, ketones and lactones have been described to date ([5] and reference within). Early studies proposed γ-decalactone as the major contributor to peach aroma with smaller contributions from other volatiles such as C6 aldehydes and terpenoids [6]. Another lactone, γ-jasmolactone, which has a peach-like odor, has been reported in handmade peach juice [7]. Other lactones found in peach: γ-octalactone, γ-dodecalactone, δ-decalactone and 6-pentyl-a-pyrone [5], [8], [9], also have pleasant aroma descriptions such as “fruity” or “coconut-like” [7] and contribute to the overall aroma of peach [10].

Studies on peach volatiles have mainly been focused on the profiling of volatiles in fruits during maturity and ripening [9], [11], [12], [13], cold storage [14], and on postharvest treatments [9], [15], culture techniques and management [16] and germoplasm variability analysis [5]. Even, the distribution of the volatile compounds throughout the fruit has been studied [8].

In spite of the vast amount of data that has been gathered on peach volatile production and the organoleptic description of the main aroma-contributing compounds, nothing is known about how this complex set of volatiles is regulated. Metabolite correlation patterns, analyzed mainly using metabolomic correlation networks, are believed to provide relevant information about the underlying biological system [17], [18] and could give insight into network regulation [19], [20]. Metabolite-metabolite correlation has been used to decipher co-regulated volatile compounds in other economically important crops. For tomato, it was revealed that volatile compounds derived from the same biochemical pathway are highly correlated [21]. The interactions between volatiles and primary metabolites were analyzed by means of correlation networks [22] and hierarchical cluster analysis [23]. In melon, a combination of complementary metabolomic profiling platforms permitted the study of the association of volatile compounds with inorganic elements and primary and secondary non-volatile metabolites [24].

Gene function discovery in peach is currently being prompted by the recent release of the whole genome sequence (http://www.rosaceae.org) and the availability of genomic tools, e.g., microarrays [25], [26] and transient gene expression assays in fruit [27]. Establishing a metabolomic platform for high-throughput volatile compound profiling for peach and describing the volatile production network is a first step that will provide the groundwork that will aid future studies directed at identifying genes related to aroma formation.

In this work, we applied a non-targeted data analysis approach to describe the volatile compound complement of peach fruit. We analyzed metabolite-metabolite correlations to gain insight into the co-regulation of peach volatiles and studied the correlations with conventional fruit quality parameters to analyze those volatiles that affect peach quality. Moreover, a correlation network analysis of the complete data set revealed the interactions between different groups of volatiles (e.g. negative interaction between the lactone groups and lipid-derived volatiles). In addition, several volatile compounds that had not yet been described in peach fruit were readily assigned to co-regulated groups and/or putative metabolic pathways. These results contribute to defining the peach volatile map including the regulatory and interaction patterns which we believe will be useful for breeding or biotechnological purposes.

## Materials and Methods

### Ethics Statement

No specific permits were required for the described field studies since the authors are responsible for the fields used. Our study did not imply any national parks or protected areas.

### Experimental Design and Fruit Analysis

Peach fruits were harvested in July, 2009 from a local commercial orchard situated in Murcia, Spain (genotypes: Granada, Maruja, MxR_01 and RedCandem) and from IVIA experimental fields in Valencia, Spain (genotype: MxR_01). Granada and Maruja are clingstone non-melting peaches while MxR_01 and RedCandem are freestone melting-fleshed. Maruja is a native Spanish material. RedCandem and Granada are cultivars obtained from different breeding programs (USA and Brazilian, respectively). MxR_01 is an F1 hybrid between the RedCandem and Maruja genotypes. The fruits of all of the genotypes were harvested at the commercial maturity stage. Besides for MxR_01 and Granada genotypes, three earlier stages of maturity were also evaluated. All four genotypes were analyzed at harvest and after shelf-life conditions to cover the changes that occur during ripening under normal conditions during peach commercialization. Twenty fruits from each maturity stage (cultivar and location) were collected and sorted into 2 groups. Fruits in one group were immediately analyzed and those in the other were subjected to shelf-life simulation conditions (2 days at 20°C, 85% RH). Quality parameters were analyzed at harvest and after shelf-life simulation: peel ground colour, flesh firmness, fruit weight and soluble solids content (SSC). Peel ground colour was evaluated using a Minolta Colorimeter (Model CR-300, Ramsey, N.Y., U.S.A). L, a, and b Hunter parameters were measured, and the results were expressed as colour index (IC = 1000a/Lb). Flesh firmness was determined with an Instron Universal Machine model 4301 texturometer (Instron Corp., Canton, Mass., U.S.A.) using an 8-mm plunger, after epicarp removal, at 2 equidistant locations in the equatorial region of each fruit. Texture data were expressed as the maximum force in kgf required to break the flesh. SSC values were measured twice for each fruit with a digital refractometer (model PR1, Atago, Tokyo, Japan) and the results were expressed as °Brix. Based on the quality parameter measurements, the 3–6 fruits representative of each condition (genotype, maturity stage, shelf-life simulation, and location) were selected for volatile analysis as follows. A summary of the experimental design and analysis is presented in the supplementary data (Fig. S1).

### Sample Preparation and HS-SPME-GC-MS Conditions

Volatile analysis was performed essentially as described in Tikunov et al. [21] with minor modifications. Immediately after the firmness measurements, the peach fruits were peeled and middle mesocarp tissue samples were ground to powder in liquid nitrogen. A 500-mg sample of frozen tissue powder was weighed in a 7-ml vial, which was then sealed and incubated at 30°C for 10 min. 500 µ1 of 100 mM EDTA-NaOH (pH 7.5) solution and 1.1 g of CaCl_2_.2H_2_O were immediately added to terminate endogenous enzyme activity. The samples were agitated and sonicated for 5 min in closed vials. A 1-ml aliquot of the homogenate was then transferred into a 22-ml crimp cap vial (Perkin-Elmer), capped, and used for HS-SPME-GC-MS analysis. Volatile analysis was performed on a 6890 N Agilent gas chromatograph coupled to a 5975B inert XL MSD mass spectrometer (Agilent Technologies). Volatiles were extracted and injected automatically by means of a CombiPAL autosampler (CTC Analytics). After incubating the samples for 10 min at 50°C with continuous agitation (500 rpm), the volatile compounds were extracted by adsorption to a 65-µm polydimethylsiloxane-divinylbenzene fiber (Supelco) and placed in the head space of the vial for 10 min under the same conditions (50°C, 500 rpm). Volatiles were desorbed by direct injection into the port of the gas chromatograph for 1 min at 250°C in splitless mode. Separation was performed on a DB-5 ms column (60 m x 0.25 mm i.d., 1-µm film thickness; J&W Scientific). Helium was used as the carrier gas at a flow rate of 1.2 mL min–1. The temperature program started at 35°C for 2 min, followed by a 5°C min–1-ramp to 250°C, with a 5-min hold at 250°C. Mass spectra were obtained at an ionization energy of 70 eV and a scan speed of 7 scans s–1, with a mass-to-charge ratio scan range of 35 to 220. In a number of cases commercial standards were used to confirm the chemical nature of the predicted compounds (see below).

### Data Processing and Statistical Analysis

The Multivariate Mass Spectra Reconstruction (MMSR) approach developed by Tikunov et al. [21] was used for the non-targeted data analysis. Chromatograms were processed (automated baseline correction, mass spectra extraction and subsequent spectral data alignment) simultaneously using the dedicated MetAlign software package (http://www.metalign.nl). A peak threshold factor of 10 was chosen for the baseline correction in the MetAlign interface. The metabolic profiles aligned were subjected to Hierarchical Cluster Analysis (HCA) using Acuity software 4.0 (Axon Instruments). The Pearson correlation coefficient was used as the similarity metric and complete linkage as the linkage method for HCA. To assign molecular fragments to compounds, the HCAs were manually inspected. Those molecular fragments which revealed a Pearson correlation higher than 0.8 and eluted within a 3-s retention time window (which corresponds to the maximum peak width at one-half height observed in the chromatograms we obtained) were considered to belong to the MS spectrum of a given compound.

For compound identification, the most suitable chromatogram (i.e. the one showing a high abundance of the selected ion with no overlapping with other compounds at the specific position) was selected. Metabolites were putatively identified using the MSD ChemStation software (Agilent Technologies). When necessary, peak deconvolution was undertaken using the AMDIS software [28]. Compound name was assigned if: 1) all fragments of the cluster were present in the spectral mass of the peak and 2) the match against the NIST mass spectral library (http://www.nist.gov) was higher than 800 (with 1000 being a perfect match). A specific ion (m/z) was selected for the quantification of each compound. The ratio of the signal relative to that of a reference sample was log 2-transformed. A reference sample consisting of a mix of all samples analyzed during the experiment was included daily in order to correct fiber aging and the temporal variation of the system.

The Acuity 4.0 software (Axon Instruments) was also used for metabolite hierarchical cluster analysis, heatmap visualization, principal component analysis and Pearson correlation evaluation.

For metabolomic network construction, the ExpressionCorrelation plug-in (http://www.baderlab.org/Software/ExpressionCorrelation) for the Cytoscape software [29] was used. High and low cut-offs of the Pearson correlation coefficient of 0.6 and −0.6, respectively, were selected. The network was visualized using the Cytoscape software v2.7.0 (www.cytoscape.org).

## Results

### Identification and Relative Quantification of Peach VOCs by a Non-targeted Data Analysis Approach

In order to obtain an exhaustive description of the volatile compound complement of the peach fruit, a set of quite diverse samples was analyzed. The sample set comprises a total of 85 samples covering different peach types, diverse genetic origins, different locations, maturity stages and physiological responses as is described in the Materials and Methods section (Fig. S1). The specific results describing the details of this phenotypic variation (genetic background, environmental effect, maturity stage and shelf-life simulation) are beyond the scope of this article and will be presented elsewhere.

The Multivariate Mass Spectra Reconstruction (MMSR) approach was used for the non-targeted data analysis for peach volatile identification and relative quantification. A total of 102 GC-MS chromatograms (85 samples, 12 external references and 5 blanks) were analyzed with the MetAlign software. After baseline correction, a total of 6,316 molecular fragments were obtained and aligned across the 102 GC-MS datasets. As a first step, the 6,316 molecular fragments were subjected to HCA to identify contaminant-derived molecular fragments. Contaminant-derived molecular fragments were recognized by being present in blank samples. Fragments coming from the SPME fiber material showed typical polysiloxane ions (e.g., m/z 207, 208, 73, etc.). These molecular fragments were excluded from the dataset.

After preprocessing, the dataset was resubjected to HCA for metabolite identification. A total of 110 clusters (candidate compounds) of molecular fragments were recognized. HCA inspections identified 90 as being putative VOCs, while 20 remained unidentified (Ni, Table S1). The reliability of our identification procedure was 100% confirmed in a set of 53 volatiles for which we have commercially available standards (Table S1). The structure and mass spectra for the 90 putatively identified VOCs (including those confirmed with standards as labeled by an asterisk) are presented as supplemental data (Fig. S2).

In order to assess the success of our pipeline, several chromatograms were inspected manually in order to obtain an estimate of the total metabolite number. A list of 124 volatiles was recognized manually, indicating that the approach was able to discover around 89% (110∶124) of the total VOCs present in our experiment.

Our complex experimental setup confers several advantages upon our VOC study: it enables the characterization of a more complete volatile compound complement than previous approaches and increases the robustness of the metabolite-metabolite correlation analysis, since different variability sources are analyzed.

### Peach VOCs are Clustered According to their Biochemical and/or Structural Nature

**Figure 1:**
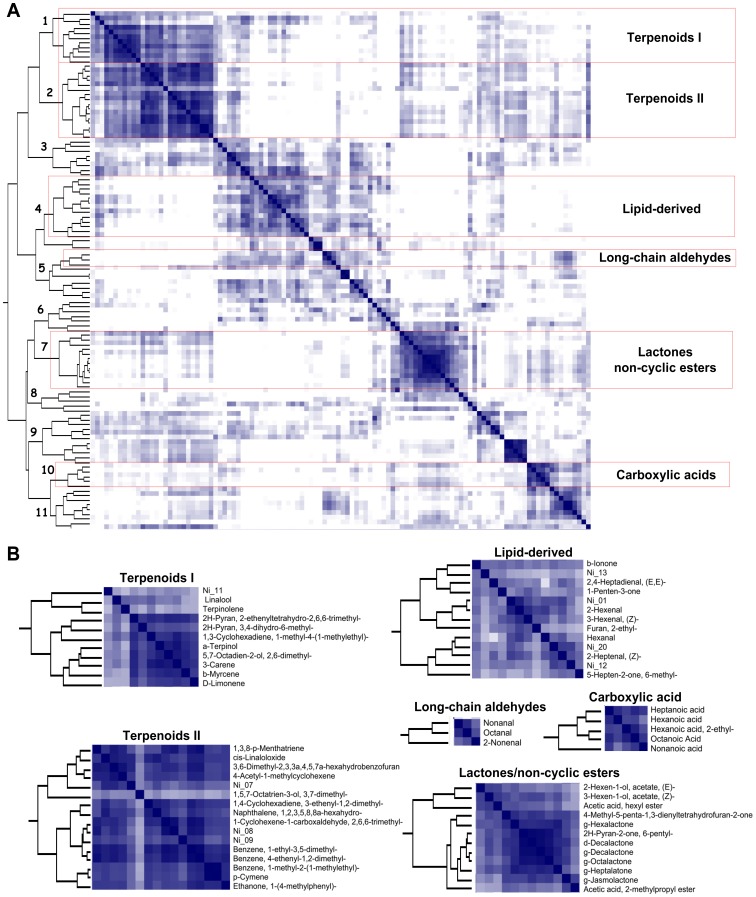
Hierarchical cluster analysis and heatmap of metabolite-metabolite correlation matrix. A) Metabolite clusters (1–11) are indicated. The heatmap was constructed using positive Pearson correlation coefficients. The darker the colour, the higher the correlation coefficient. B) Detail of the main clusters showing the metabolite members. The compounds: γ-Jasmolactone, γ-Hexalactone, γ-Heptalatone, γ-Octalactone, γ-Decalactone, δ-Decalactone, δ-Limonene, β-Myrcene and β-Ionone are indicated in the figure as: g-Jasmolactone, g-Hexalactone, g-Heptalatone, g-Octalactone, g-Decalactone, d-Decalactone, D-Limonene, b-Myrcene and b-Ionone, respectively.

In order to explore the metabolite-metabolite relationships in peach fruit, the entire data set of 9,350 elements (85 samples × 110 metabolites) was subjected to HCA. The HCA revealed that the volatiles can be organized into different clusters, indicating a tight metabolite regulation (Fig. 1A). As is shown in Fig. 1B, each cluster mainly contains compounds with similar chemical structures (i.e., lactones, non-cyclic esters, carboxylic acid and long-chain aldehydes) or belong to specific metabolic pathways (i.e., lipid-derived metabolites and terpenoid biosynthesis).

**Figure 2:**
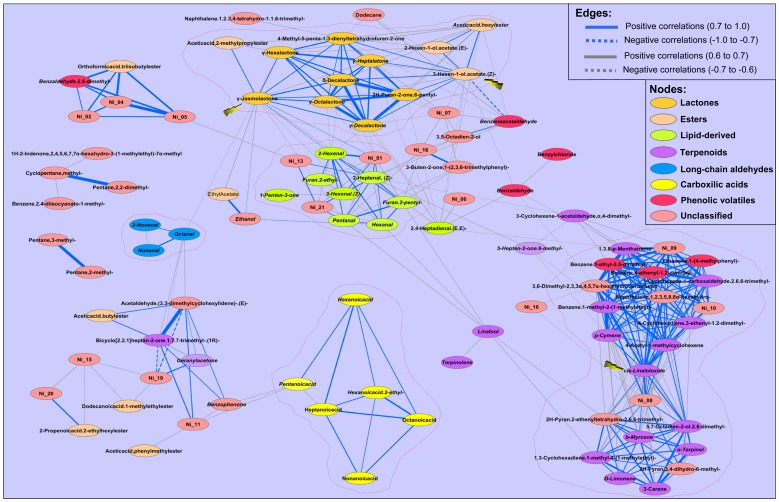
Metabolomic correlation network analysis. The main clusters identified in Fig. 1 are highlighted with a dotted red line. Nodes are codified with colours according to their chemical nature or their belonging to known metabolic pathways described in other plant species. Correlations lower than 0.7 in absolute value are drawn in grey. Correlations between 0.7–1.0 (absolute value) are in blue. Line thickness indicates correlation strength: the wider the line, the stronger the correlation. Positive correlations are represented by straight lines, negative correlations by dotted lines. The yellow arrows highlight the metabolites that have a central role in the metabolomic network (3-Hexen-1-ol acetate, (Z)-, γ-Jasmolactone and cis-Linaloloxide); for a detailed description see the text. The compounds: γ-Jasmolactone, γ-Hexalactone, γ-Heptalatone, γ-Octalactone, γ-Decalactone, δ-Decalactone, δ-Limonene, and β-Myrcene are indicated in the figure as: g-Jasmolactone, g-Hexalactone, g-Heptalatone, g-Octalactone, g-Decalactone, d-Decalactone, D-Limonene and b-Myrcene, respectively.

**Figure 3:**
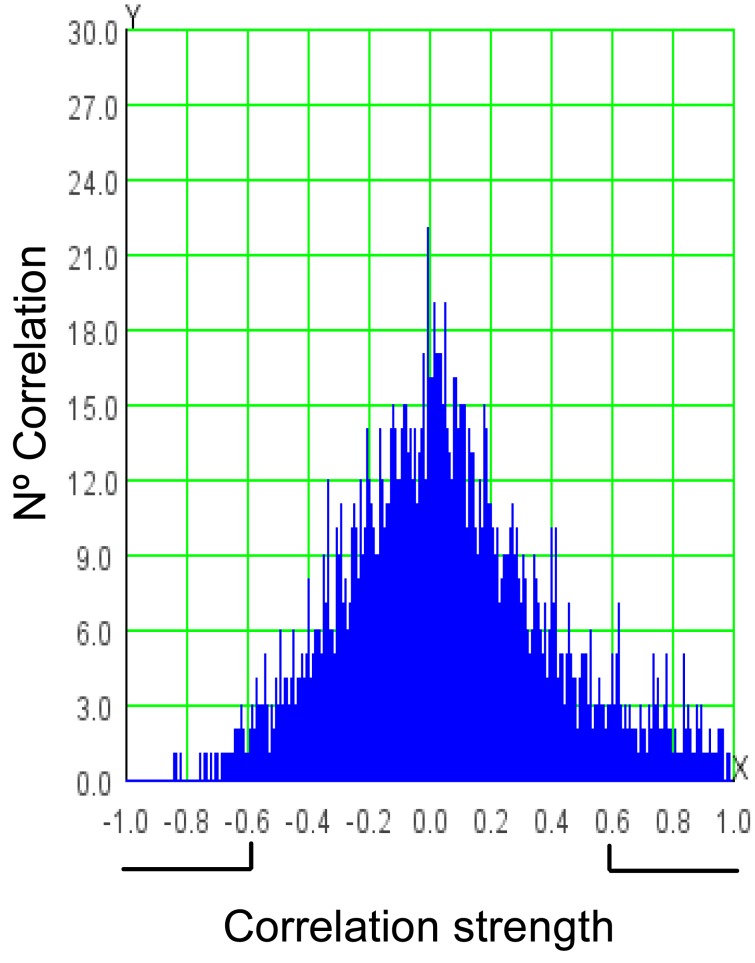
Distribution of the correlations evaluated. Black lines below the graph show the correlations taken to construct the network.

To further explore new metabolite-metabolite correlations and metabolic group interactions, we conducted a network correlation analysis using the complete data set (Fig. 2). A correlation network was constructed using the Pearson correlation coefficient. The histogram of the 5,995 metabolite-metabolite correlations evaluated for all possible VOC pairs is shown in Fig. 3. In order to simplify the graphic, only strong correlations (r>0.6 in absolute value) were represented in this network. The metabolic correlation network obtained consists of 96 nodes (VOCs) and 374 edges (correlations, 326 positive and 48 negative). As expected, the volatile clusters observed in the HCA could be readily identified in the correlation network as groups of highly interconnected metabolites (Fig. 2). Lactones (γ-Hexalactone, γ-Heptalactone, γ-Octolactone, γ-Decalactone, δ-Decalactone, 2HPyran-2-one 6-pentyl, 4-Methyl-5-penta-1, 3-dienyltetrahydrofuran-2-one and γ-Jasmolactona) showed high positive correlations between one another. Interestingly, these cyclic esters (lactones) are connected to some linear esters (Acetic acid hexyl ester, 2-Hexen-1-ol acetate (E) and 3-Hexen-1-ol acetate (Z)-) mainly through 3-Hexen-1-ol acetate (Z), which suggests an interaction between their biosynthetic pathways. In addition, γ-Jasmolactone levels were highly correlated (r = 0.90) with Acetic acid 2 methyl propyl ester (described as a fruity VOC) and showed a moderate correlation (r = 0.68) with ethanol and its ester (ethyl acetate) which are normally associated with the over-ripening process [30]. Similarly, lipid-derived volatiles (Pentanal, Hexanal, 2-Hexanal, 3-Hexanal (Z)-, 2-Heptenal (Z)-, Furan 2-pentyl and Furan 2-ethyl) and 3 unidentified VOCs (Ni_01, Ni_12 and Ni_20) form a highly interconnected group. This group of volatiles is inversely correlated with the lactones/ester clusters principally through γ-Jasmolactone.

Terpenoid compounds mostly grouped into two highly correlated clusters linked through cis-linaloloxide.

The long-chain aldehydes (Octanal, Nonenal and 2-Nonenal) and carboxylic acids (Pentanoic acid, Hexanoic acid, Hexanoic acid 2-ethyl, Heptanoic acid, Octanoic acid and Nonanoic acid) remain as groups weakly related to other volatile groups.

On the other hand, correlation network analysis revealed the grouping of compounds that were not related structurally or by any known biochemical pathway. This is the case of Orthoformic acid tri-isobutyl ester and Benzaldehyde 2,5-dimethyl-, which are highly correlated with a small group of unidentified compounds (Ni_02, Ni_03, and Ni_04, Fig. 2). In addition, two terpenoid compounds (Geranyl acetone and Bicyclo[2.2.1]heptan-2-one 1,7,7-trimethyl- (1S)-), one ester (Acetic acid butyl ester) and four unclassified compounds (Ni_18, Ni_10, Benzophenone and Acetaldehyde (3,3-dimethylcyclohexylidene)- (E)-) were shown to be interconnected by positive and negative correlations (Fig. 2), as will be discussed further on.

Finally, our correlation network analysis also identified a set of volatiles (i.e., Pentane, 2-methyl and Pentane, 3-methyl) that are apparently not related to any other compound of the volatile complement.

### Some Volatile Compounds can be Associated to Quality Traits

In addition to the description of the relationship between compounds in the peach volatile complement, correlations with quality parameters were studied in order to identify VOCs putatively associated with a good, ripe peach. As a number of quality traits, such as ground colour, fruit weight and SSC, increase during ripening according to genotype, VOCs showing positive correlations to these traits could positively influence fruit quality. Similarly, fruit firmness decreases during ripening, and therefore VOCs associated with this fruit quality trait should be inversely correlated to this parameter. To find robust correlations, we analyzed Pearson correlation coefficients between a series of quality parameters and VOCs for the entire 85-sample set. VOCs that showed the strongest correlations with regard to ground colour (Table 1), flesh firmness (Table 2), fruit weight (Table 3) and SSC (Table 4) are shown. All lactones identified in our materials (8 in all) were highly correlated with ground colour (r = 0.72 to 0.85), and 7 also showed strong negative correlations with firmness (r = 0.75 to 0.88 in absolute value). Some lactones (γ-Hexalactone, γ-Heptalatone and γ-Jasmolactone) also showed correlations with weight (r = 0.69, 0.57 and 0.55, respectively), but with lower values as compared to colour and firmness. A number of esters were highly correlated with quality parameters: Ethyl acetate showed a strong correlation with colour (r = 0.67), 3-Hexen-1-ol acetate (Z)- and Acetic acid hexyl ester were inversely correlated with firmness (r = −0.84 and −0.73, respectively) and 2-Hexen-1-ol acetate (E)- was correlated with weight (r = 0.63). In general, correlations with weight were lower (r = 0.54 to 0.69) than those with colour (r = 0.67 to 0.85) and firmness (r = 0.73 to 0.88 in absolute value). Nevertheless, weight also showed correlations with other volatiles besides lactones and esters. Although they were significant, volatile compounds showed low correlations with SSC (r = 0.25 to 0.52).

**Table 1:** Correlation analysis between volatiles and ground colour.

Ground colour	
**VOCs**	**+PCC (r)**
γ-Jasmolactone	0.85
δ-Decalactone	0.79
γ-Decalactone	0.77
γ-Octalactone	0.76
4-Methyl-5-penta-1,3-dienyltetrahydrofuran-2-one	0.76
γ-Heptalatone	0.74
2H-Pyran-2-one, 6-pentyl-	0.73
γ-Hexalactone	0.72
Ethyl Acetate	0.67
**VOCs**	**−PCC (r)**
Ni_06	**−**0.71
Ni_01	**−**0.67
2-Hexenal	**−**0.63
Furan, 2-pentyl-	**−**0.61
Ni_17	**−**0.59
2-Heptenal, (Z)-	**−**0.58
Pentanal	**−**0.58
Eugenol	**−**0.56
Benzeneacetaldehyde	**−**0.54

Volatiles showing the strongest positive and negative correlations are shown. +PCC, Positive Pearson correlation coefficient; **−**PCC, Negative Pearson correlation coefficient. The correlations shown are significant (α<0.05).

**Table 2:** Correlation analysis between volatiles and flesh firmness.

Flesh firmness	
**VOCs**	**−PCC (r)**
γ-Decalactone	**−**0.88
δ-Decalactone	**−**0.87
2H-Pyran-2-one, 6-pentyl-	**−**0.85
3-Hexen-1-ol, acetate, (Z)-	**−**0.84
4-Methyl-5-penta-1,3-dienyltetrahydrofuran-2-one	**−**0.81
γ-Heptalatone	**−**0.81
γ-Octalactone	**−**0.77
γ-Hexalactone	**−**0.75
Acetic acid, hexyl ester	**−**0.73
**VOCs**	**+PCC (r)**
2,4-Heptadienal, (E,E)-	0.76
Ni_06	0.73
Benzeneacetaldehyde	0.69
Dodecane	0.65
2-Heptenal, (Z)-	0.64
Ni_01	0.62
3,5-Octadien-2-ol	0.59
2-Hexenal	0.56
Benzaldehyde	0.56

Volatiles showing the strongest positive and negative correlations are shown. +PCC, Positive Pearson correlation coefficient; **−**PCC, Negative Pearson correlation coefficient. The correlations shown are significant (α<0.05).

**Table 3:** Correlation analysis between volatiles and fruit weight.

Fruit weight	
**VOCs**	**+PCC (r)**
γ-Hexalactone	0.69
Ethanone, 1-(4-methylphenyl)-	0.67
2-Hexen-1-ol, acetate, (E)-	0.63
1,3,8-p-Menthatriene	0.60
Benzene, 4-ethenyl-1,2-dimethyl-	0.58
γ-Heptalatone	0.57
1,5,7-Octatrien-3-ol, 3,7-dimethyl-	0.55
γ-Jasmolactone	0.55
3-Cyclohexene-1-acetaldehyde, α,4-dimethyl-	0.54
**VOCs**	**−PCC (r)**
2-Heptenal, (Z)-	**−**0.73
Benzeneacetaldehyde	**−**0.60
Ni_20	**−**0.57
Hexanal	**−**0.56
3-Hexenal, (Z)-	**−**0.55
3,5-Octadien-2-ol	**−**0.55
Ni_01	**−**0.54
2-Hexenal	**−**0.54
Dodecane	**−**0.53

Volatiles showing the strongest positive and negative correlations are shown. +PCC, Positive Pearson correlation coefficient; **−**PCC, Negative Pearson correlation coefficient. The correlations shown are significant (α<0.05).

**Table 4:** Correlation analysis between volatiles and SSC.

SSC	
**VOCs**	**+PCC (r)**
3-Hexen-1-ol, acetate, (Z)-	0.52
3-Buten-2-ol, 2-methyl-	0.43
Acetic acid, hexyl ester	0.36
Hexanal	0.29
γ-Heptalatone	0.28
2H-Pyran-2-one, 6-pentyl-	0.27
γ-Decalactone	0.26
Terpinolene	0.26
δ-Decalactone	0.25
**VOCs**	**−PCC (r)**
1H-2-Indenone,2,4,5,6,7,77α-hexahydro ….^a^	**−**0.69
Ni_14	**−**0.57
Ni_06	**−**0.53
Dodecanoic acid, 1-methylethyl ester	**−**0.45
2,4-Heptadienal, (E,E)-	**−**0.45
Benzeneacetaldehyde	**−**0.44
1,5,7-Octatrien-3-ol, 3,7-dimethyl-	**−**0.43
3-Buten-2-one, 1-(2,3,6-trimethylphenyl)-	**−**0.40
Benzaldehyde	**−**0.39

Volatiles showing the strongest positive and negative correlations are shown. +PCC, Positive Pearson correlation coefficient; **−**PCC, Negative Pearson correlation coefficient. The correlations shown are significant (α<0.05).

a The full name of the compound is: 1H-2-Indenone,2,4,5,6,7,7α-hexahydro-3-(1-methylethyl)-7α-methyl.

Volatiles showing inverse correlations with colour, weight and SSC, or direct correlations with firmness, could be involved in immature fruit aroma and could therefore have a negative impact on peach quality. In general, lipid-derived volatiles and a number of unidentified compounds associated with them (Fig. 1 and Fig. 2) showed moderate-to-high correlations with quality parameters (Tables 1, 2, 3, 4). This is the case of 2-Hexenal, Furan 2-pentyl-, 2-Heptenal (Z)-, Pentanal and Ni_01, which showed an inverse correlation with colour values (r = 0.58 to 0.67 in absolute value). Regarding firmness, 2,4-Heptadienal (E,E)- showed the strongest correlation (r = 0.76) as did 2-Heptenal (Z)- and Ni_01, although these had significant but lower coefficients (r = 0.64 and 0.61, respectively). In the case of weight, it is strongly but inversely correlated to 2-Heptenal (Z)-, and moderately to lowly correlated with Hexanal, 3-Hexenal (Z)- and 2-Hexenal (Table 3). Other volatiles unrelated to lipid metabolism were also correlated with quality parameters. For example, Ni_06 showed a high correlation with colour (r = **−**0.71) and firmness (r = 0.73), and Benzeneacetaldehyde was correlated with all quality parameters, with values ranging from r = 0.44 to 0.69 (in absolute value). In our study, SSC was poorly correlated with volatile compounds with the exception of 1H-2-Indenone 2,4,5,6,7,7α-hexahydro-3-(1-methylethyl)-7α-methyl, which showed an inverse correlation of r = **−**0.69 (Table 4).

## Discussion

### Description of the Peach Fruit Volatile Compound Complement Using a Non-targeted Approach

A combination of analytical technique (HS-SPME-GC-MS), chromatogram aligment tools (MetAlign) and data analyses (cluster analysis and VOC identification) was used to profile the volatile compound complement of a complex sample set of peach fruit and to reveal the network of metabolic VOC interactions. A similar approach was previously used to analyze VOCs in tomato fruit, and it was estimated that approximately 80% of the total tomato VOCs were detected [21]. Here, 89% of the total number of volatile compounds present in our sample set were identified, indicating the power of our platform.

HS-SPME is considered an easy and inexpensive extraction method when compared to liquid–liquid extraction, solid-phase extraction (SPE), vacuum distillation or dynamic headspace. Nevertheless it has been poorly used to profile peach VOCs with some exceptions. Using HS-SPME-GC-MS, Wang et al. studied fruit VOCs of a peach germplasm collection; by analyzing 50 peach and nectarine fruits of different origins, they identified 84 VOCs [5]. We have putatively indentified a similar number of volatile compounds in this report (90, Table S1). For a group of 53 VOCs, we have confirmed the robustness of our identification procedure by using commercial standards, which had a 100% success rate. Additionally, our approach resulted in the identification of several compounds that had not been reported previously in peach fruit (e.g., 34, 39, 44, 58, 67, 88 and 97, numbered according to Table S1 and Fig. S2). All of this supports that the approach used here was able to profile a substantial range of the peach volatile compound complement.

We also studied correlations between VOCs and the main quality parameters of the fruit in order to describe volatiles putatively involved in ripe peach aroma (Tables 1, 2, 3, 4). Peach quality is directly affected by the maturity state of the fruit as well as the conventional parameters used to characterize the fruit maturity stage (peel ground colour, flesh firmness, soluble solid content, weight and titratable acidity). The change in peel ground colour (from green to orange-red, increasing colour index values) is a common criterion used to harvest the fruit at the right time in order to obtain high quality peaches. SSC and weight also rise during ripening while fruits accumulate sugars and increase in size, respectively. Contrary, flesh firmness decreases during fruit ripening as a result of the natural softening of the fruit. Thus, VOCs contributing to ripe peach aroma and therefore to peach quality are expected to be directly correlated with colour, weight and SSC and inversely correlated with flesh firmness. In order to identify more robust relationships (rather than genotype-specific ones), we took advantage of our complex sample set which includes different genotypes, developmental and ripening stages and some postharvest variants, and evaluated the correlations between volatiles and fruit quality parameters throughout all 85 samples (Tables 1, 2, 3, 4). Lactones showed strong correlations with colour (positives values) and flesh firmness (negatives values), suggesting that they are associated with fruit qualities assayed by these parameters irrespective of the genotype. Lactones have been previously found to be involved in fruit ripening [12] and have been reported as being main contributors to mature fruit aroma in peach [6], [7], [10]. The lactone putatively identified as 4-Methyl-5-penta-1,3-dienyltetrahydrofuran-2-one has not yet been described in peach, although it appears to be the most abundant volatile in certain species of chanomeles fruit [31], which belongs to a different genus within the *Rosaceae* family. The fact that this compound showed high correlations with quality parameters (r = 0.76 with colour and r = **−**0.81 with firmness) suggests that it could be involved in ripe peach aroma. Nevertheless, in order to accurately evaluate the involvement of this compound in overall peach aroma, the odor quality as well as the odor activity and threshold values need to be analyzed.

Correlation analysis also indicated that lipid-derived compounds (2-Hexenal, Furan, 2-pentyl-, Pentanal, Hexanal, 3-Hexenal, (Z)-, 2-Heptenal (Z)- and 2,4-Heptadienal (E,E)-) could be related to immature fruit (Tables 1, 2, 3, 4). Lipid-derived compounds have been traditionally described as “green” aromas, and in the case of 2-Hexenal and 3-Hexenal, (Z)- they were described as confering “green” notes to handmade peach juice [7]. In our study, the volatile compound 2,4-Heptadienal, (E,E)- showed the highest correlation with flesh firmness (r = 0.76), suggesting that it accumulates in immature fruit. The odor of 2,4-Heptadienal (E,E) has consistently been described as “green/fatty” (www.thegoodscentscompany.com). This aldehyde is found in plum and apricot cultivars [32], but had so far not been described in peach fruits. Our study revealed a number of compounds (e.g., Ni_01 and Ni_06) that might also be involved in immature aromas of peach, but their structure remains to be elucidated.

In summary, although additional work is required to establish the precise and definitive nature of the link between traits and volatile metabolites, the information described in this paper could serve as a starting point.

### Volatile Complement of Peach Fruit Reveals a Highly Organized Network

The ultimate goal of a metabolomics approach is to provide an unbiased identification and quantification of all metabolites present in a system to then describe it in a comprehensive and rational manner [33]. In this study, we described the volatile compound complement of the peach metabolome and analyzed the metabolite-metabolite correlation to gain insight into co-regulated groups of volatile compounds, some of which may directly affect fruit quality.

In order to accurately evaluate metabolite-metabolite correlations, it is necessary to analyze a system showing consistent variation in the variables under study. Genetic variation can be used as a source for phenotypic VOC variation which can be analyzed in an attempt to reveal the underlying metabolic networks. In a previous study, ripened fruits from a large set of commercial tomato cultivars were analyzed in this way [21]. The same strategy was also used in *Citrus* to obtain and analyze phenotypic variation [34]. Recently, an alternative strategy was reported for evaluating metabolite-metabolite correlation using different fruit parts and development stages of melon fruit [24], as opposed to genotypic variation, in an attempt to obtain modifications of the metabolite network. Here, we present a combination of these two strategies by analyzing samples from different genetic backgrounds and maturity stages while also including different geographical locations and physiological responses (Fig. S2). By combining diverse origins of variation, which affect the phenotypic VOC variation, our study proposes an alternative strategy to using the MMSR approach described previously to evaluate metabolite-metabolite correlations. This alternative method could be particularly useful in cases where a large number of genotypes are unavailable, as is usually the case for fruit crops or wild species, or when a larger number of perturbations of the metabolic network are needed to increase the power of the approach.

In our study, the two types of correlation analysis (HCA and metabolic network analysis) revealed that most of the compounds clustered according to their biochemical nature: lactones, non-cyclic esters, carboxylic acids and long-chain aldehydes. In some cases, they clustered according to their known metabolic pathways as described in other species, i.e., lipid-derived metabolites and terpenoid biosynthesis (Fig. 1; Fig. 2). The relationship between the metabolic correlation network and the underlying biochemical pathways has been studied previously using GC-TOF/MS-derived data as a model [18], [19], [35]. Currently, it is generally accepted that although correlation patterns cannot be directly extrapolated to biochemical pathways, they can still be very informative about the underlying system [19]. Since metabolic pathways involved in the synthesis of volatile compounds in peach are still poorly known, we have restricted our analysis in terms of pragmatic fruit quality implications. Nevertheless, we can still provide empirical data that confirms known volatile biochemical pathways operating in peach fruit and suggest some novel ones. For example, in some fruits, acetaldehyde can be interconverted into ethanol by the alcohol deshydrogenase enzyme (ADH, EC 1.1.1.1) or to acetyl coenzyme A (CoA) by the aldehyde dehydrogenase enzyme (ALDH, EC 1.2.1.5) [30]. Acetyl-CoA could then undergo subsequent reactions with alcohols to generate the corresponding acetate esters. Indeed, the high correlation between ethanol and ethyl acetate (r = 0.94) observed in the metabolic correlation network could represent one of these reactions (Fig. 2). Camacho et al. suggested several mechanisms to explain the correlation between metabolites in replicated experiments [19]. These authors stated that two metabolites in chemical equilibrium will show a high correlation across multiple experimental conditions [19]. This assertion therefore suggests that putative alcohol acyltransferases (AAT, EC 2.3.1.84) would catalyze a reversible reaction using ethanol and acetyl-CoA as the substrate in peach. Ethyl acetate has been described as having a fruity aroma [36]. Thus, putative AATs become a target of great interest for molecular engineering or plant breeding as they make it possible to obtain enzymes with the chemical equilibrium displaced towards ethyl acetate production.

Metabolic pathways leading to lactone synthesis have not yet been described in plants. However, it is commonly accepted that lactone biosynthesis in fruit starts with unsaturated fatty acids that subsequently suffer the introduction of an O atom to form hydroxy fatty acids, which in turn undergo β-oxidation leading to the 4- or 5- hydroxy acids that, after intramolecular esterification, produce the corresponding lactones [37]. By infiltrating an artificial, radiolabeled epoxy acid into fruits, it was demonstrated that nectarines (peach glabrous mutation) are able to produce lactones from epoxy acids [38]. Based on that study, it was proposed that in nectarine (or peach), the introduction of the hydroxyl groups is achieved by fatty acid epoxidation and the subsequent hydrolysis of the compound formed by epoxide hydrolases resulted in the proper hydroxy acid [38]. Here, we have shown that lactone production in peach fruit is highly correlated and lactones are key to the interactions with other volatile groups, including linear esters (Fig. 1 and Fig. 2). It has been proposed that the correlation network largely reflects biochemical regulation [35]. Thus, our results suggest that the lactone biosynthesis in peach fruit may be highly co-regulated, which is encouraging for future biotechnological and breeding/improvement efforts to identify regulatory genes that control the production of this family of compounds. Indeed, during the preparation of this manuscript, it was proposed that lactone production during the postharvest ripening of peach is regulated by the first enzyme of β-oxidation (Acyl-CoA oxidase) [39], which agrees with our hypothesis.

Ethyl acetate, in addition to 3-hexen1-ol acetate, 2-hexen1-ol acetate and acetic acid 2-methyl propyl ester, have been described as having a fruity odor [7], [36]. Our volatile metabolic correlation network indicated that 3-hexen1-ol acetate, 2-hexen1-ol acetate and acetic acid hexyl ester could belong to a co-regulated group, whereas ethyl acetate and acetic acid hexyl ester seem to be regulated independently (Fig. 2). These findings suggest the existence of at least three potential ways to improve the fruity notes of peach aroma by acting at the level of each of the groups of volatiles. Moreover, the fact that the lactone group interacts with esters (3-hexen1-ol acetate, 2-hexen1-ol acetate and acetic acid 2-methyl propyl ester) suggests that lactonization may also involve common enzymes or intermediates that would be shared with the esters. Although our results require additional experiments to confirm the cause-effect relationship, this information may prove to be very useful for breeding.

It was previously demonstrated that volatile compounds derived from the catabolism of linoleic acid through the lipoxygenase pathway, the so-called lipid-derived volatiles, clustered together in tomato [21] and in *Citrus* species [34]. Here we demonstrated that in peach, lipid-derived compounds (2,4-Heptadienal, (E,E)-, 1-Penten-3-one, 2-Hexanal, 3-Hexanal (Z), Furan 2-ethyl, 2-Heptenal (Z)- and Hexanal), along with other unidentified compounds (Ni_01, Ni_12 and Ni_20), are grouped in the same cluster (Fig. 1), and that this group negatively interacts with the lactone group (Fig. 2). Since lactone biosynthesis also start from fatty acid precursors [37], this interaction may suggest that a metabolic shift of lipid metabolism during ripening reorganizes the peach aroma from the “green notes” conferred by hexenal, 2-hexanal and 3-hexanal towards the “fruity”, “floral” or “peach-like” aromas that are characteristic of lactones. Targeting the genes or regulators of this metabolic shift may be an important biotechnological target for peach aroma modulation.

The long-chain aldehydes, octanal, nonanal and 2-nonenal, could confer an unpleasant aroma since their odor quality has been described as “fatty/tallowy” [7]. Our observation that these compounds correlated to form a close group that was poorly linked to other volatiles group (Fig. 2) allows us to predict that an effort to lower these metabolite levels would have little effect on the other aroma compounds. Similarly, our correlation network analysis revealed cis-linaloxide as a hub for the terpenoid cluster (Fig. 2), and we therefore propose the enzymes leading to its formation or metabolism as potential targets for terpenoid manipulation in peach.

Most of the strongest correlations in our analysis were positive (direct), with a few exceptions being negative (inverse) correlations, e.g., between γ-Jasmolactone and 2-Hexenal or Ni_01 (Fig 2.). The correlation between volatiles and primary metabolites in tomato fruit were recently studied using the correlation network approach and demonstrated that most of the robust correlations were direct relations [22]. It appears that this property is characteristic of the metabolomic analysis approach, although the causes of this are not yet understood.

Even though correlation analysis revealed that most of the compounds were clustered according to their chemical structure or their belonging to known metabolic pathways (Fig. 1 and Fig. 2), we also found groups of compounds with no obvious trend. This is the case of the group formed by Geranyl acetone, Bicyclo[2.2.1]heptan-2-one 1,7,7-trimethyl- (1S)-, Acetic acid butyl ester, Ni_19, Ni_11, Benzophenone and Acetaldehyde (3,3-dimethylcyclohexylidene)-(E)- (Fig. 2). It is unclear what the origin of the correlations found between these compounds could be, but it is of interest to note that Acetaldehyde (3,3-dimethylcyclohexylidene)- (E) is the active principle of commercial pheromone formulations (PAN pesticide database: www.pesticideinfo.org), which are usually used in peach orchards for insect control. Although we cannot confirm the origin of this metabolite, we cannot exclude the possibility that compounds not derived from the peach metabolism were detected in our analysis. Since our study revealed that peach-derived volatiles are highly correlated to each other (Fig. 2), a correlation network analysis could also allow the identification of contaminant volatile compounds that, because of their foreign origin, would be poorly connected to the whole metabolome. In addition, we anticipate that the metabolic correlation network may be used to study the effect of chemical treatments (applied pre- or postharvest) on peach volatile production and therefore on the final quality of the product.

In summary, although our results and those referenced in the text suggest that some of these volatiles may be co-regulated, additional work is required to prove that this is the case in peach fruit.

We have limited our interpretation to the pragmatic application of our results of volatiles that may affect aroma production, based on previous work in the peach literatures. Nevertheless, the metabolic correlation map established here could eventually aid in defining potential biotechnological targets to improve other characters (e.g., nutraceutical quality of the fruit or plant-insect communication) as novel functions for peach volatiles are discovered.

In summary, a high-resolution and sensitive platform for volatile compound analysis of peach fruit was established. This approach allowed us to identify a significant part of the volatile complement of the peach fruit metabolome. Exploring metabolite-metabolite correlations, a highly organized volatile metabolite network was discovered. In addition, several novel volatile metabolites, not described in peach fruit to date, were identified and assigned to co-regulated metabolite groups and/or putative metabolic pathways. We propose the metabolic network established here as a framework for future genetic, physiological and environmental analysis of peach volatile production as well as an important resource for improving the volatile composition in peach breeding programs.

## Supporting Information

Figure S1 Experimental design and analysis. A) Genotypes and maturity stages analyzed (S1–S4). For each genotype, four representative fruits at commercial maturity stage (S4) are shown. Valencia and Murcia indicate that fruits at S4 of MxR_01 genotype were analyzed in two locations. B) The post-treatment applied. C) Fruit and volatile organic compound analysis. HS-SPME-GC-MS: Head Space-Solid Phase Microextraction-Gas Chromatography-Mass Spectroscopy.
(PDF)

Figure S2 **Chemical structure and mass spectra of volatiles identified in peach.** All spectra were taken from the NIST/EPA/NIH mass spectral library version 2.0 with the exception of the spectra of γ-Jasmolactone (N° 100), which was obtained from the authentic standard. Compounds are numbered according to Table S1. The numbers marked with an asterisk indicate that the compound identity was confirmed with an authentic standard.
(PDF)

Table S1 **Volatile organic compounds detected in the sample set.** For each volatile, the retention time (RT) in min, the cluster (Cl) that it belongs to according to Fig. 1, the specific ion (Ion, m/z) used for quantification, the forward (F) and reverse (R) matches against the Nist library (with the exception of γ-Jasmolactone, where the F and R matches against its authentic standard are shown) and the CAS number are indicated. *compound identified by comparing its retention time with an authentic standard are highlighted in bold. ^a^The full name of compound N°91 is: 1H-2-Indenone,2,4,5,6,7,7α-hexahydro-3-(1-methylethyl)-7α-methyl. na, not available.
(PDF)
