# Supplementary figures and images for: A Non-Targeted Approach Unravels the Volatile Network in Peach Fruit
Source: PLoS One. 2012 Jun 22;7(6):e38992. doi: 10.1371/journal.pone.0038992 (PMC3382205; doi:10.1371/journal.pone.0038992)

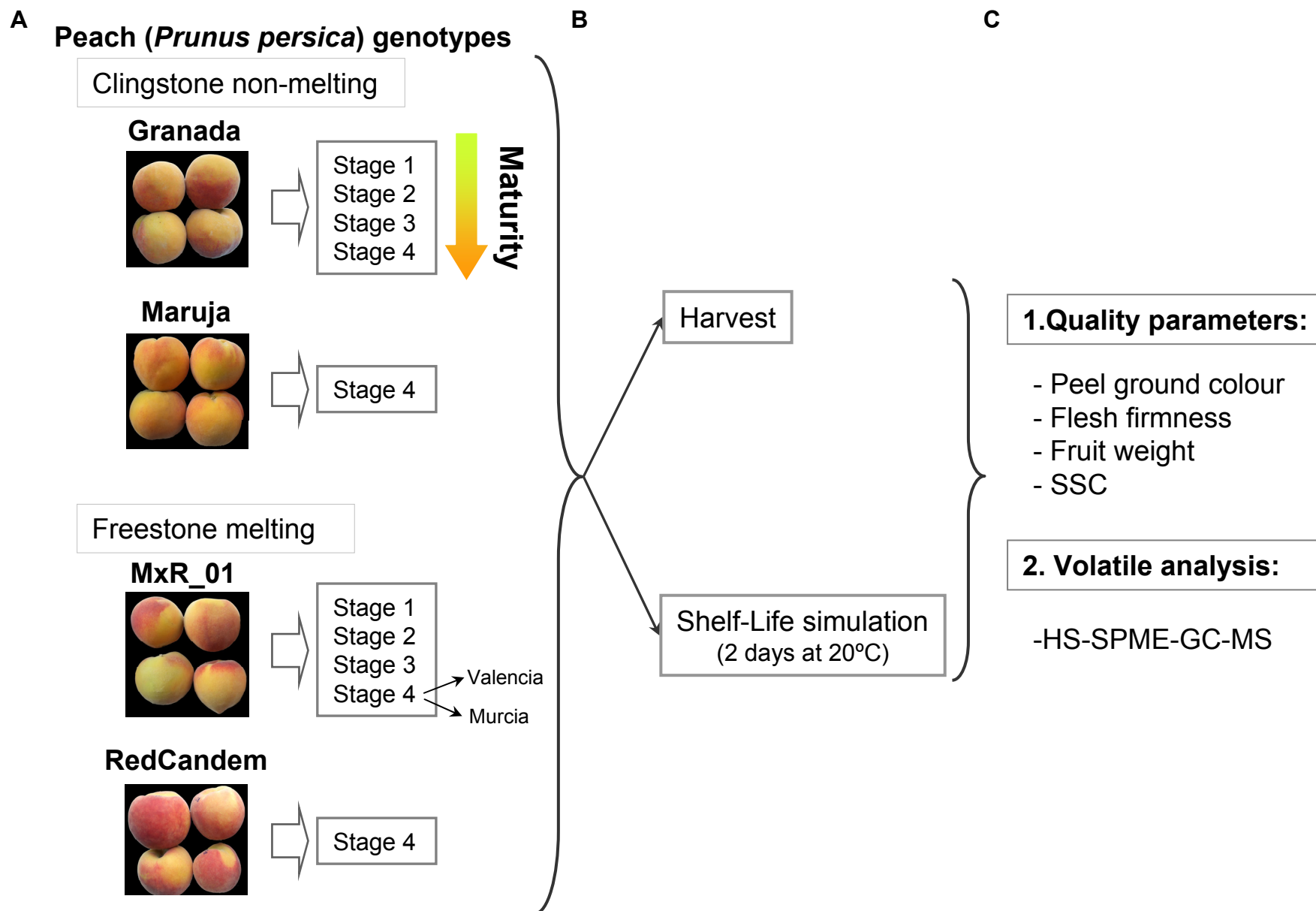

Fig. S1.

Supplement: Figure S1 — Experimental design and analysis. A) Genotypes and maturity stages analyzed (S1–S4). For each genotype, four representative fruits at commercial maturity stage (S4) are shown. Valencia and Murcia indicate that fruits at S4 of MxR_01 genotype were analyzed in two locations. B) The post-treatment applied. C) Fruit and volatile organic compound analysis. HS-SPME-GC-MS: Head Space-Solid Phase Microextraction-Gas Chromatography-Mass Spectroscopy. (PDF) [file pone.0038992.s001.pdf]
