# Supplementary material for: A Non-Targeted Approach Unravels the Volatile Network in Peach Fruit
Source: PLoS One. 2012 Jun 22;7(6):e38992. doi: 10.1371/journal.pone.0038992 (PMC3382205; doi:10.1371/journal.pone.0038992)
Supplement: Figure S2 — Chemical structure and mass spectra of volatiles identified in peach. All spectra were taken from the NIST/EPA/NIH mass spectral library version 2.0 with the exception of the spectra of γ-Jasmolactone (N° 100), which was obtained from the authentic standard. Compounds are numbered according to Table S1. The numbers marked with an asterisk indicate that the compound identity was confirmed with an authentic standard. (PDF) [file pone.0038992.s002.pdf]

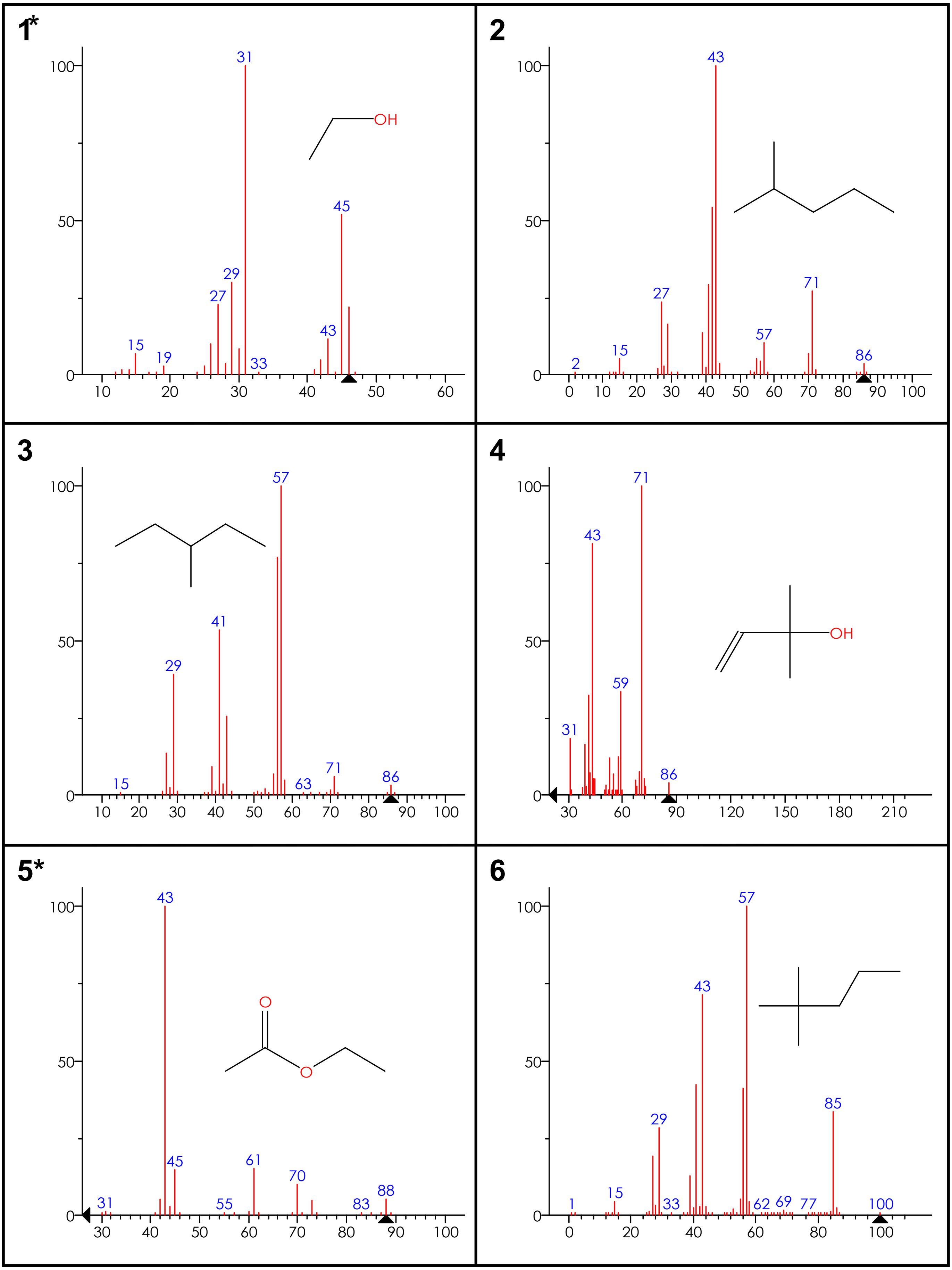

1*
31
45
29
27
15
19
43
33
OH
2
43
27
71
57
15
2
86
3
57
41
29
71
86
15
63
4
71
43
59
31
86
OH
5*
43
45
61
70
88
31
55
83
6
57
43
85
29
15
1
33
62
69
77
100

Fig. S2.

7

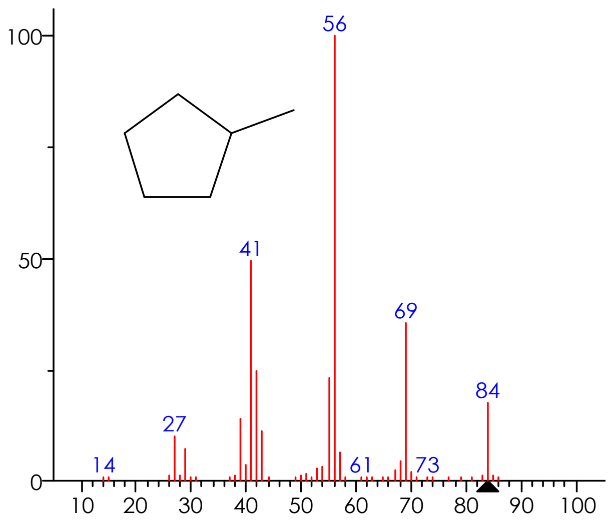

8*

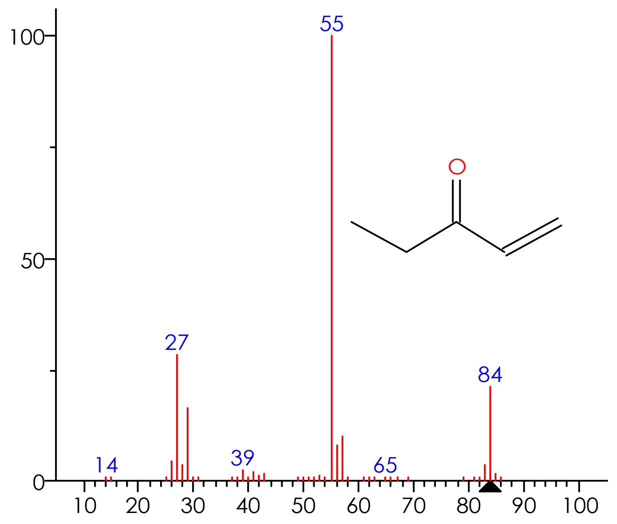

9*

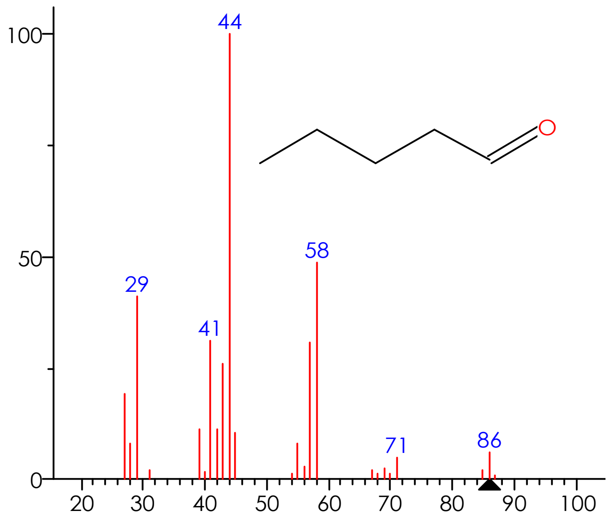

10*

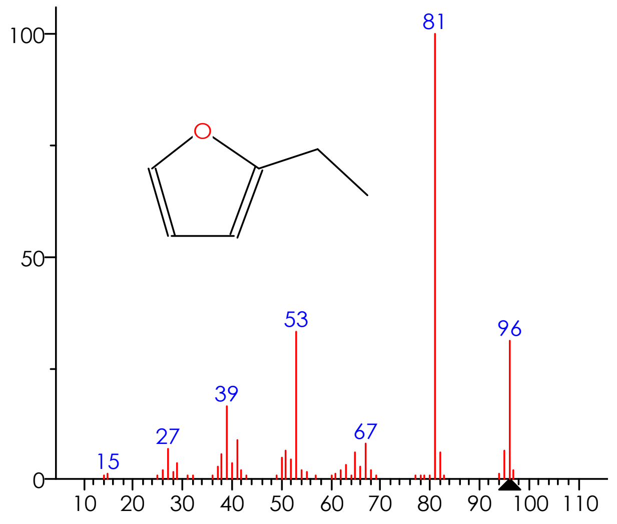

11

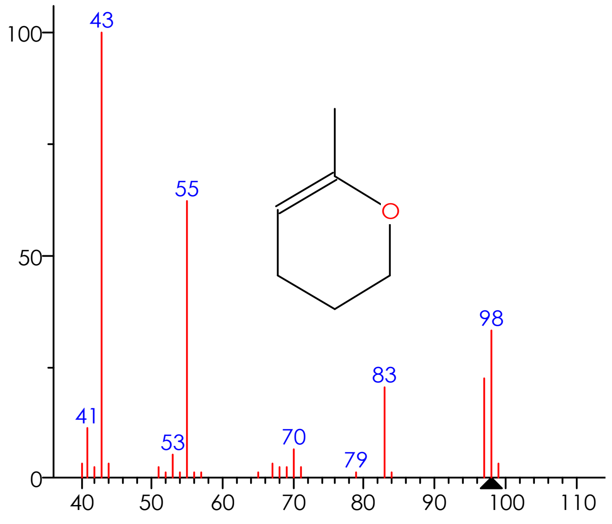

12*

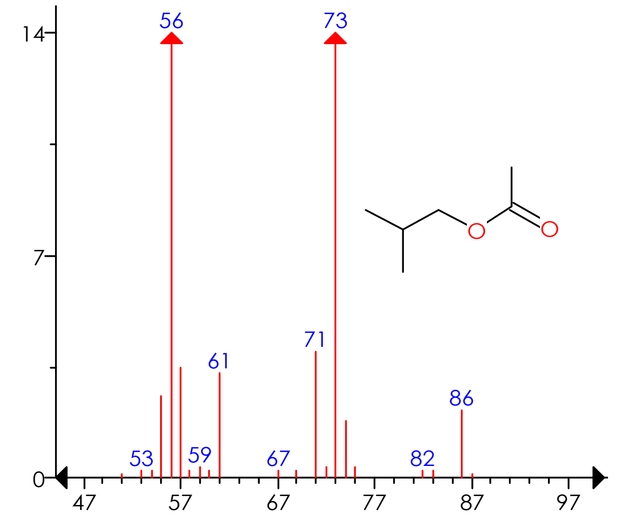

Fig. S2-continued.

13

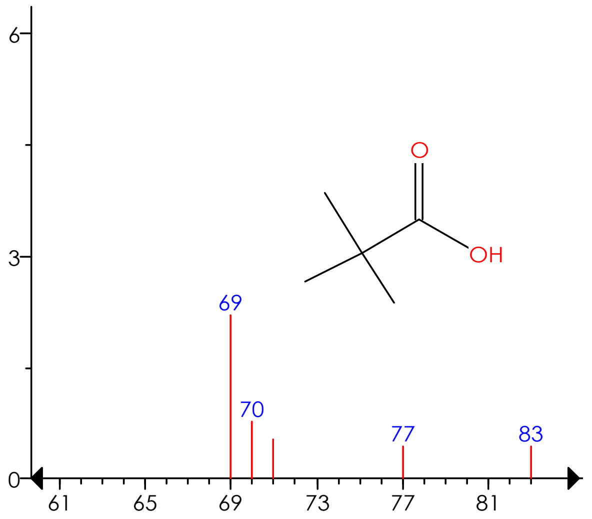

14*

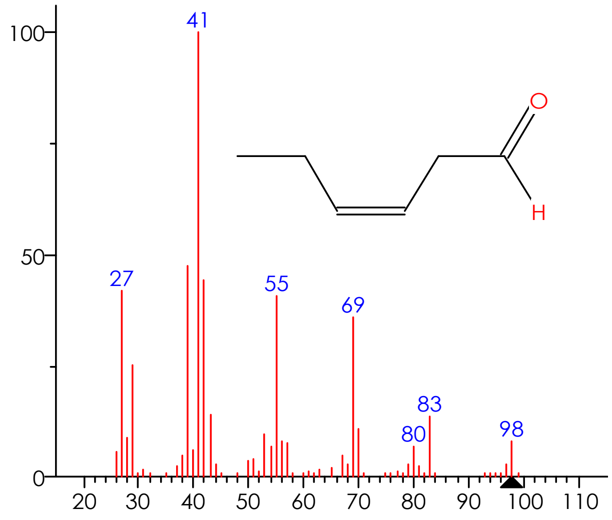

15*

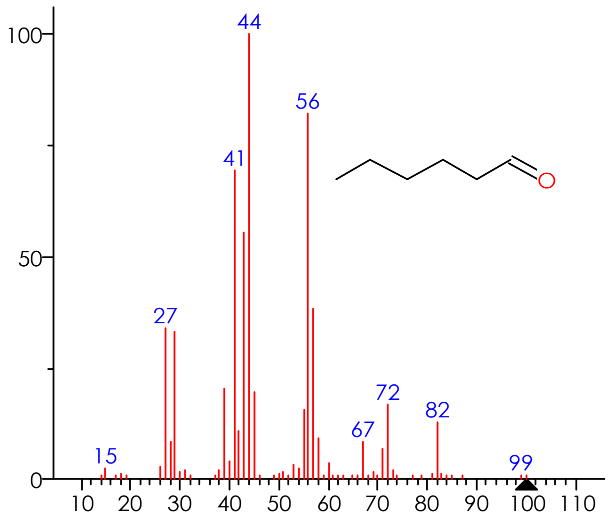

16

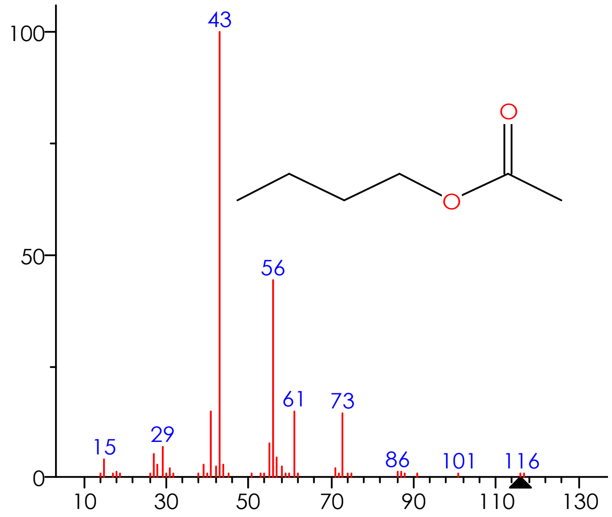

17*

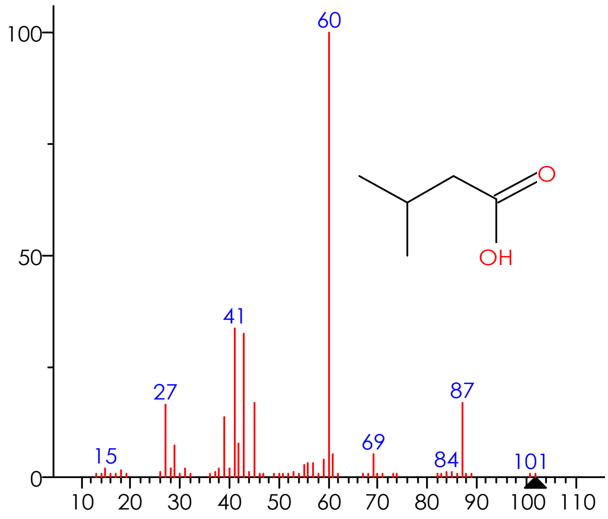

18*

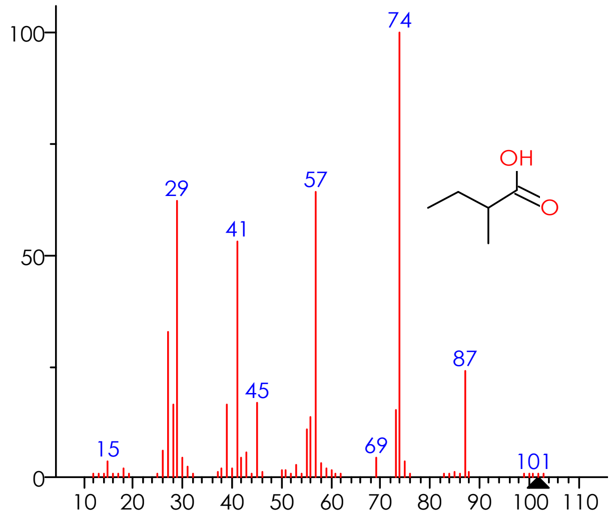

Fig. S2-continued.

20*

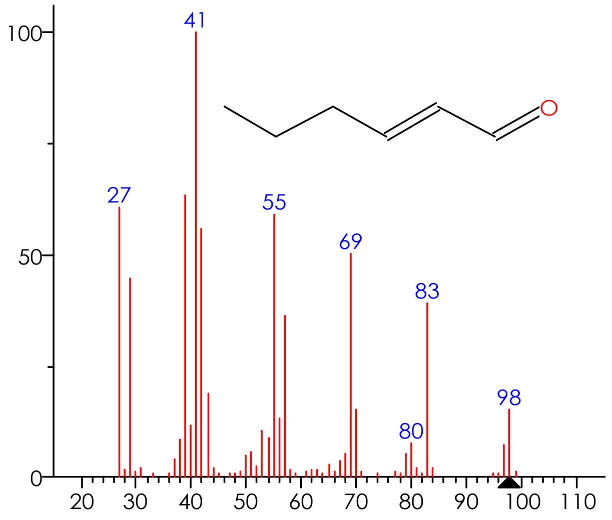

21*

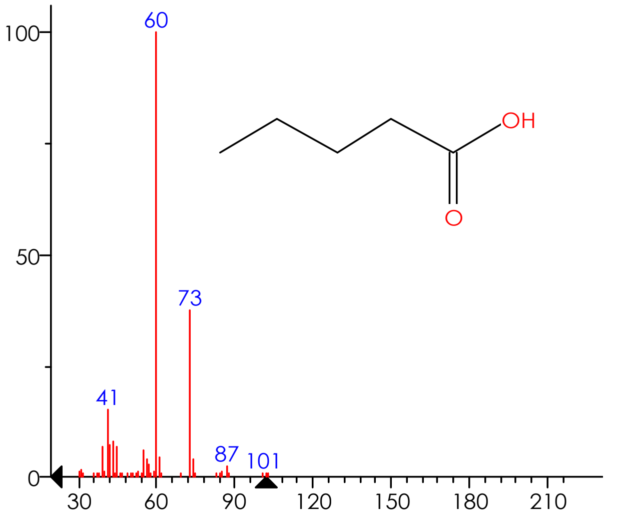

23*

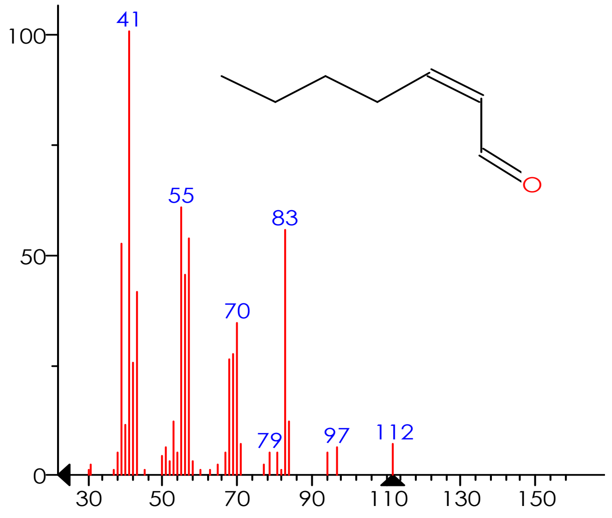

24*

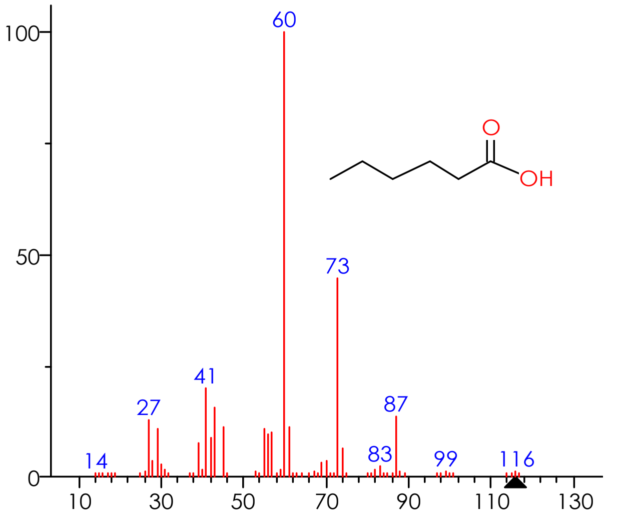

26*

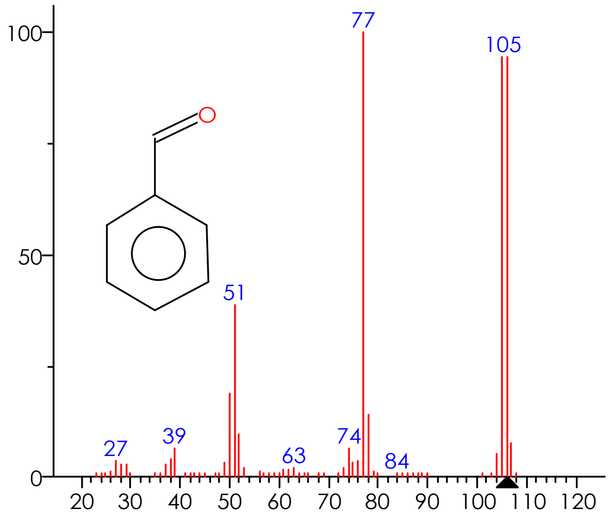

27

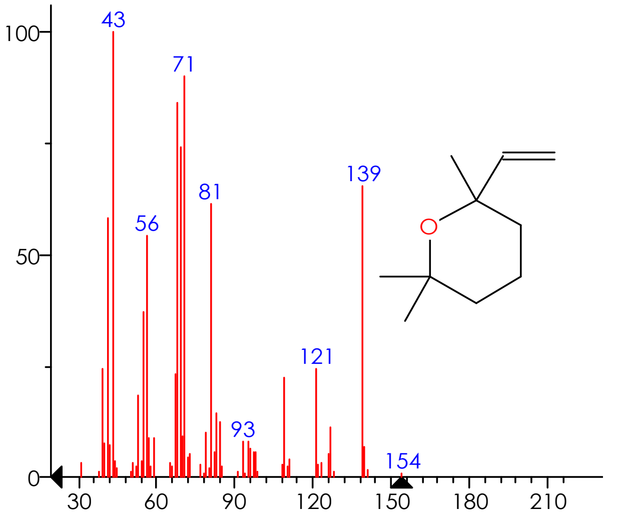

Fig. S2-continued.

28

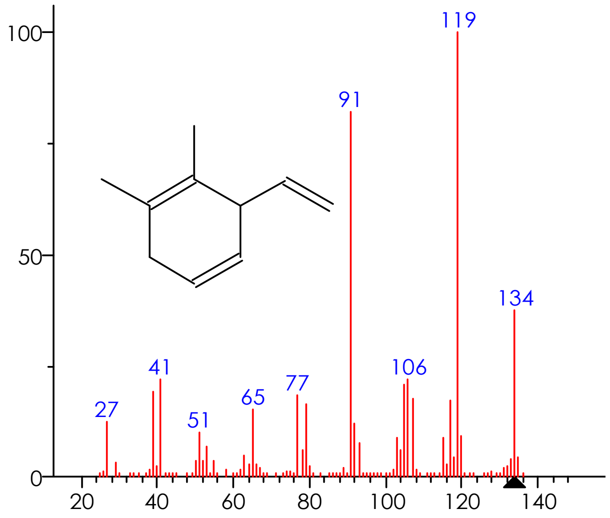

29*

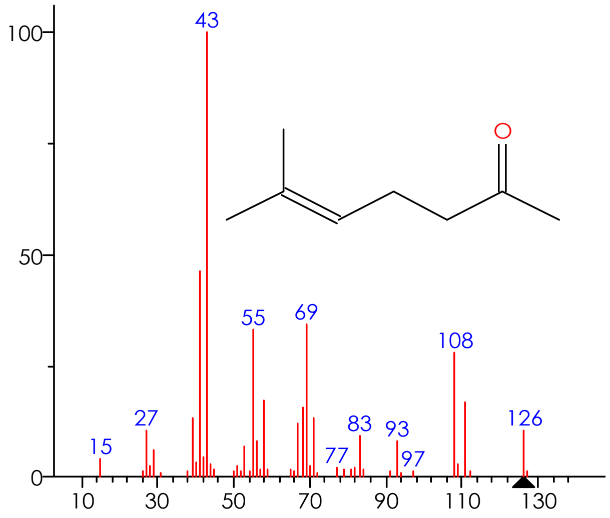

30*

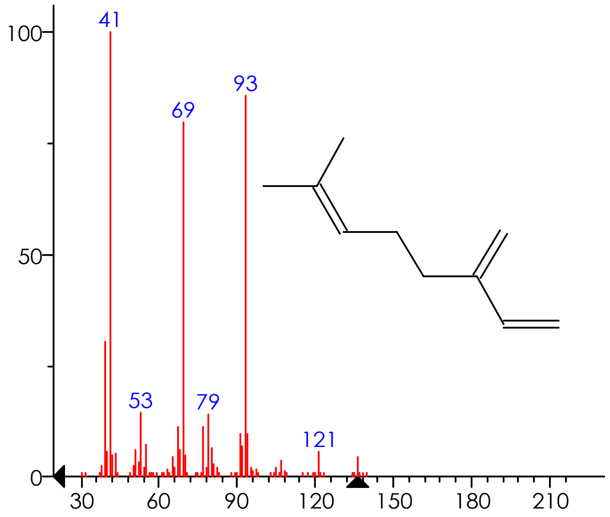

31*

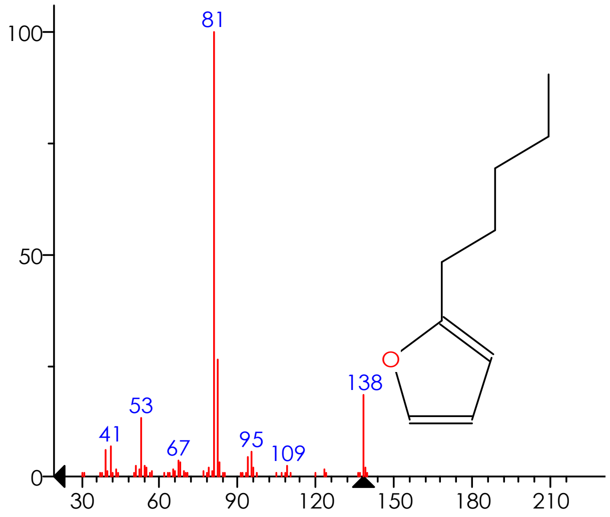

32

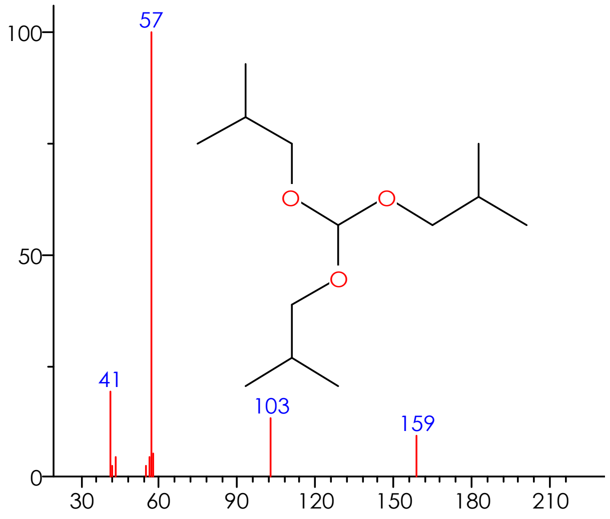

33*

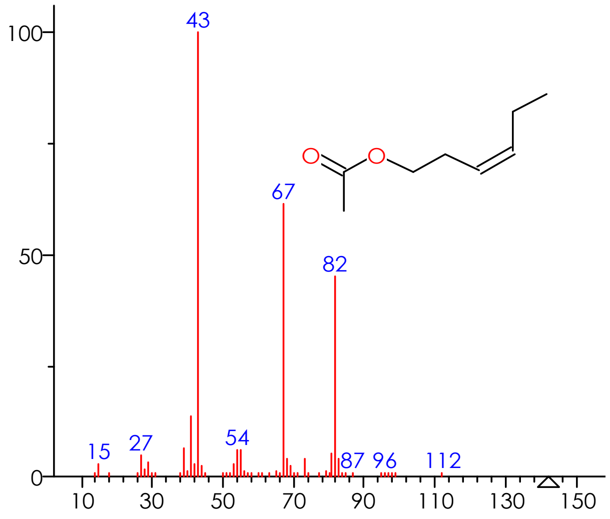

Fig. S2-continued.

34

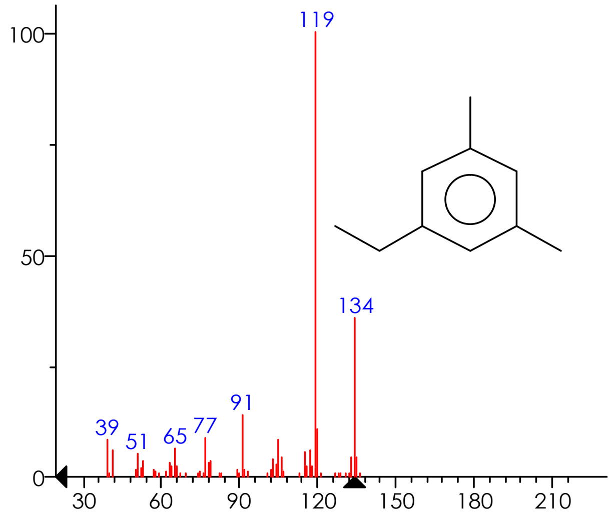

35*

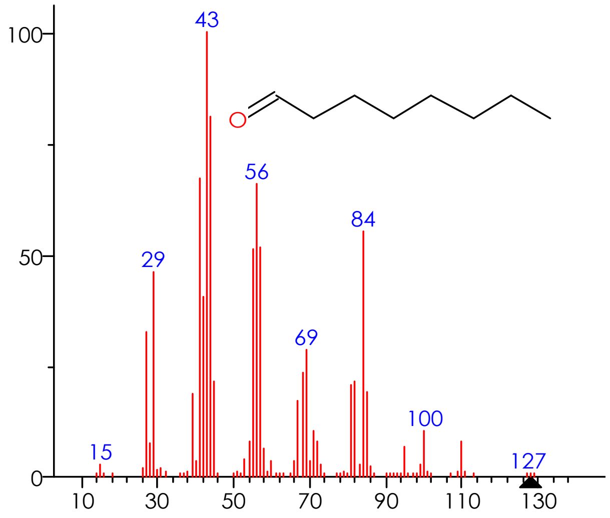

36*

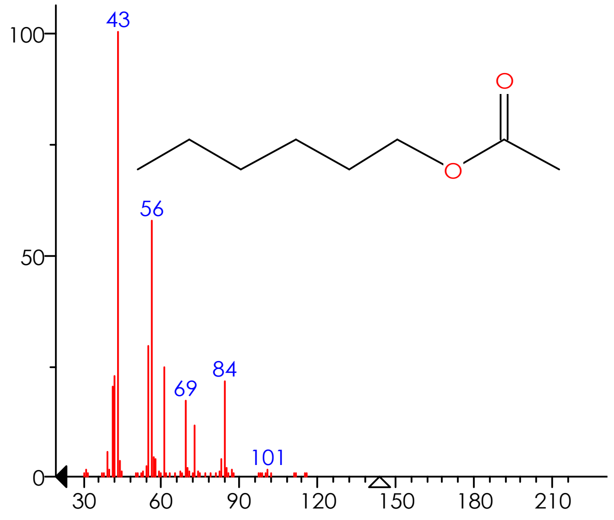

37*

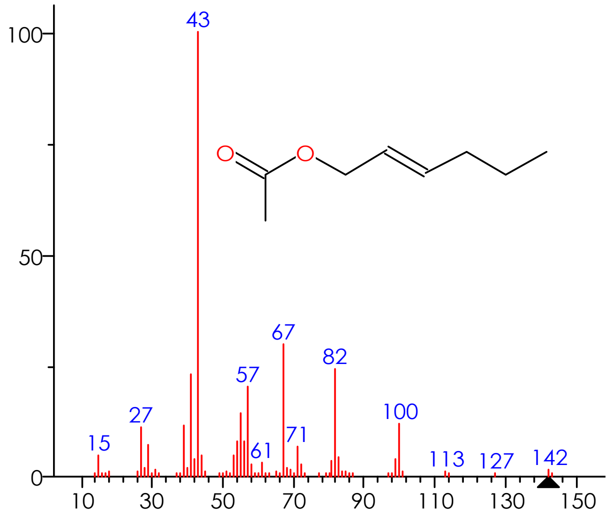

39*

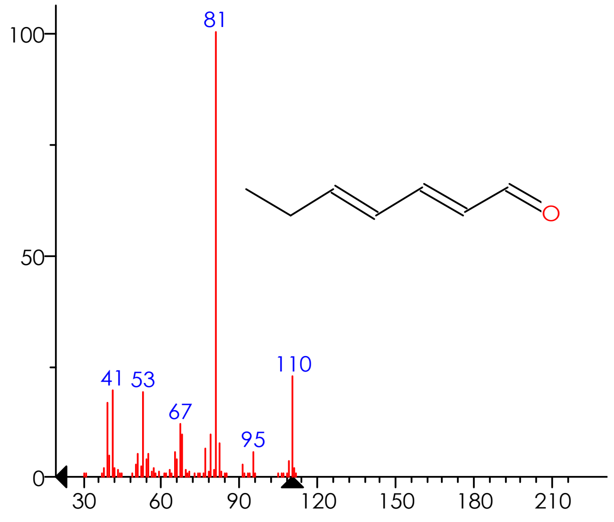

40*

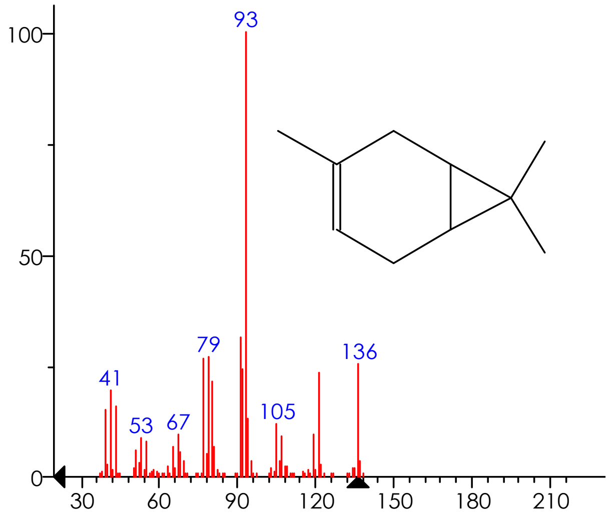

Fig. S2-continued.

41

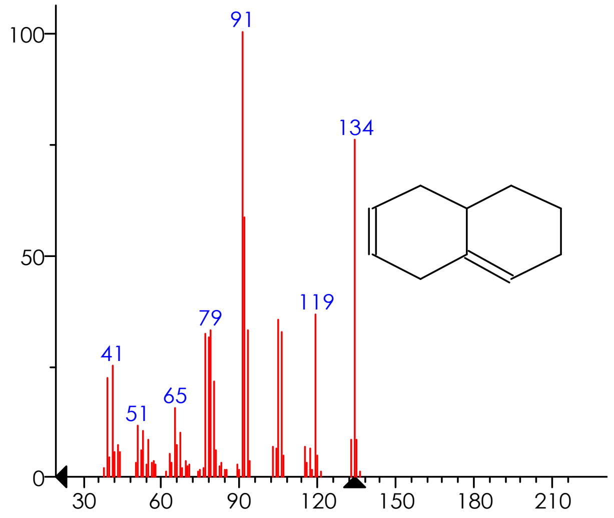

42

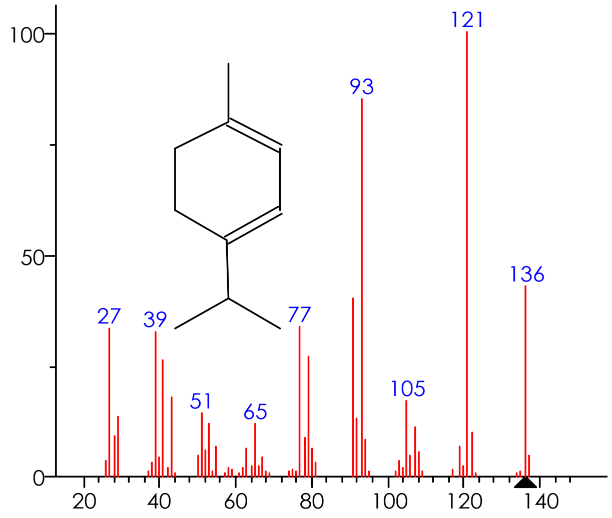

43

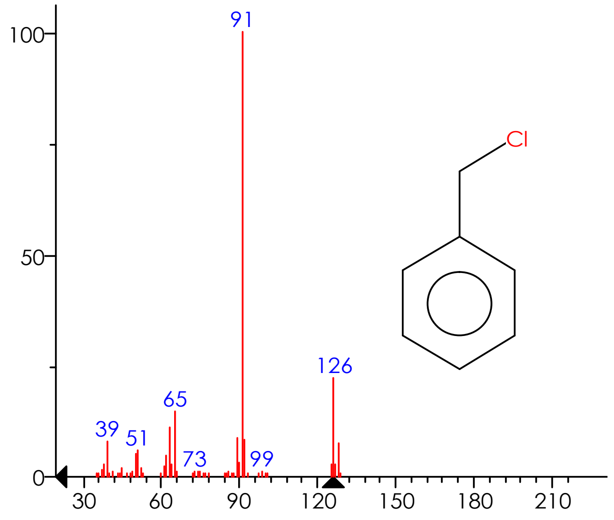

44

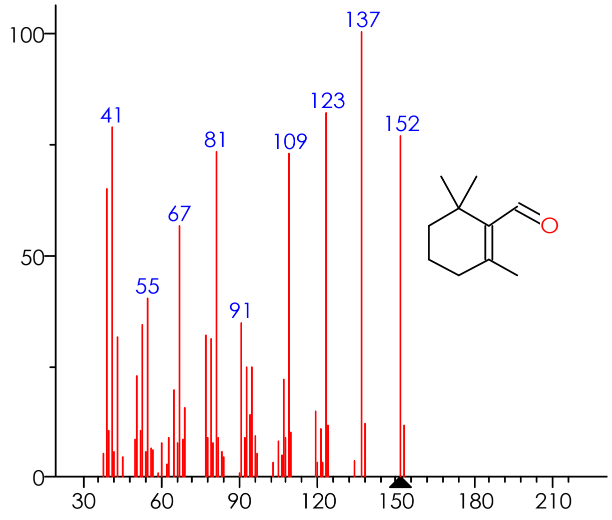

45

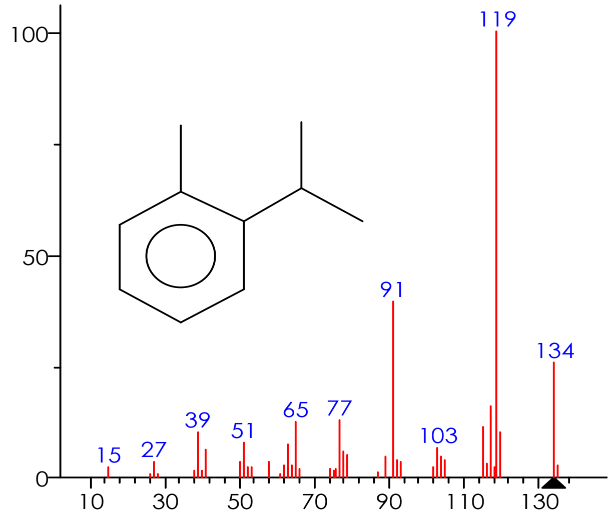

46*

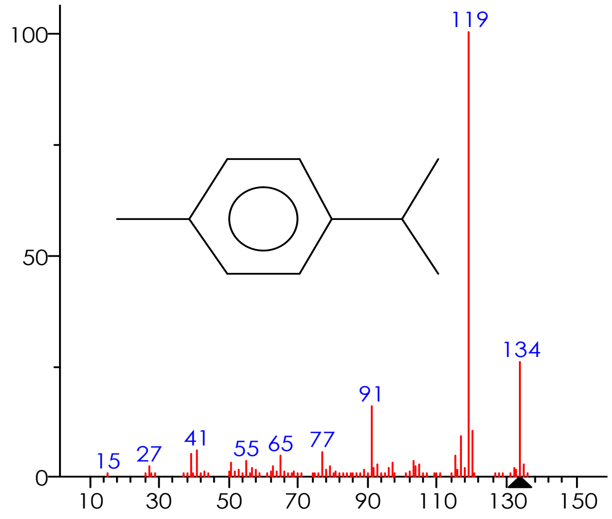

Fig. S2-continued.

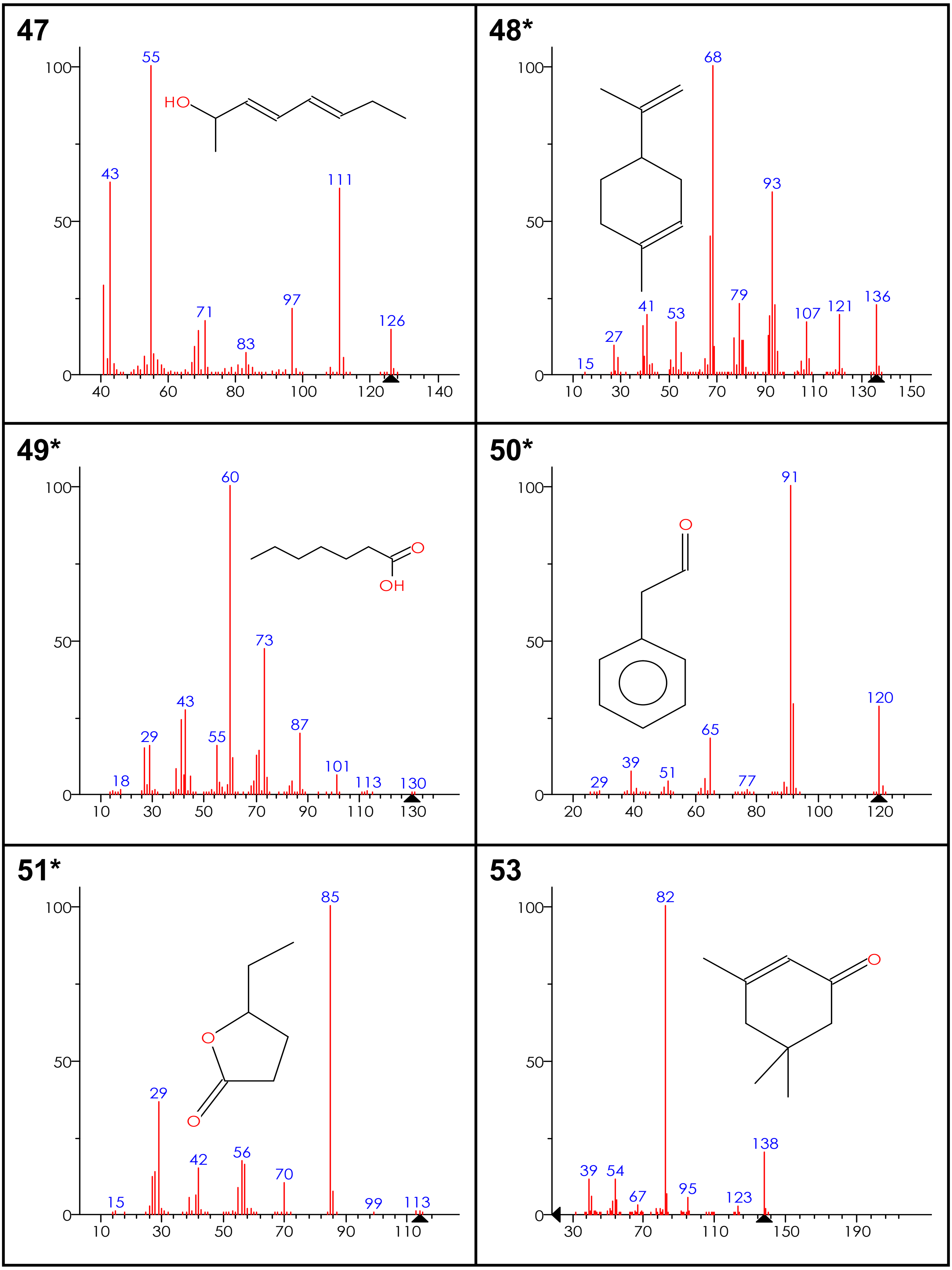

Fig. S2-continued.

54*

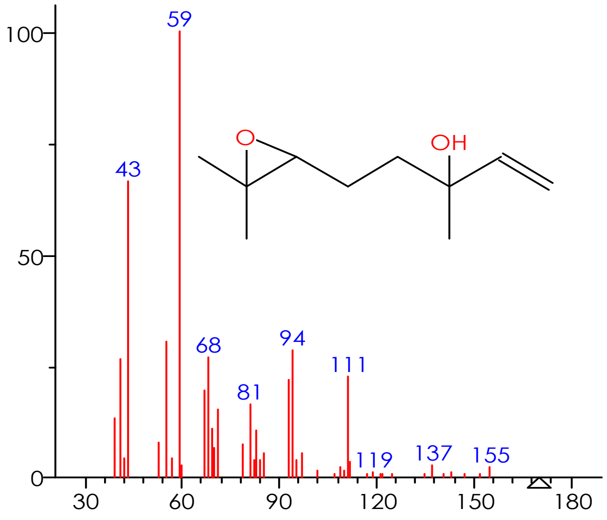

56*

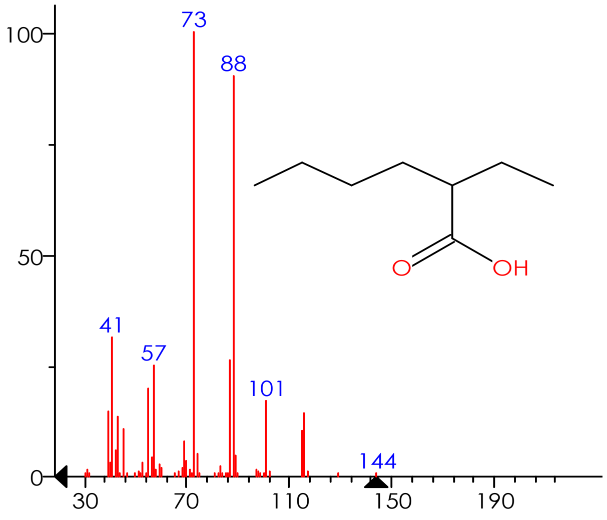

57*

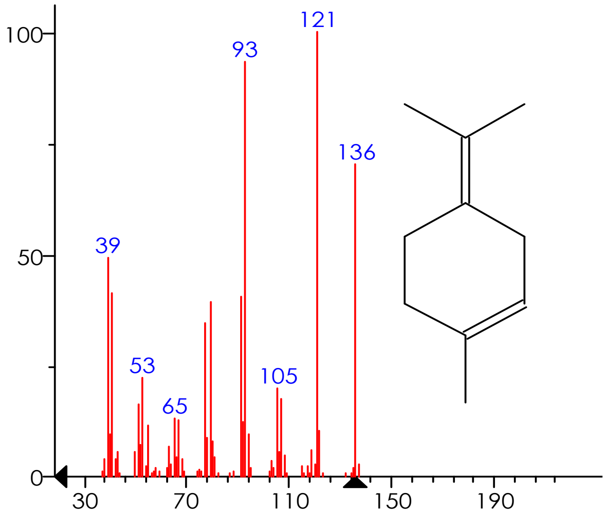

58

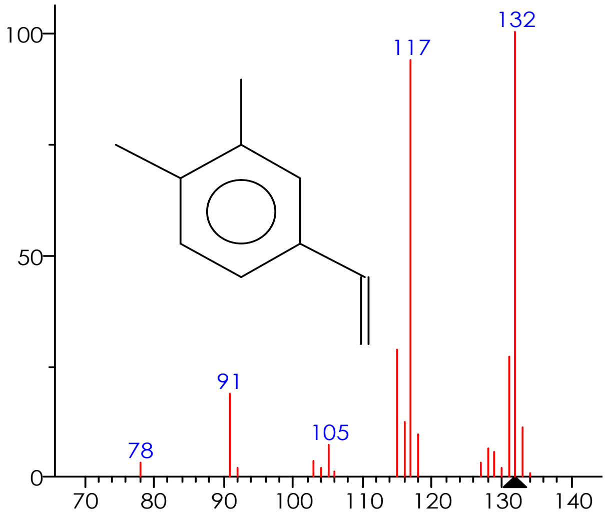

59*

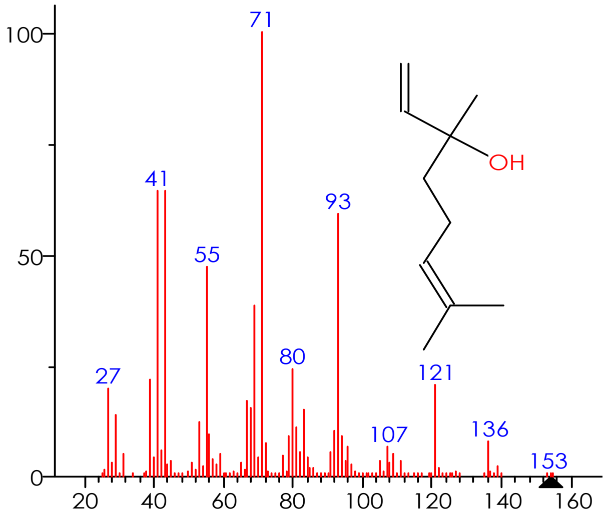

60*

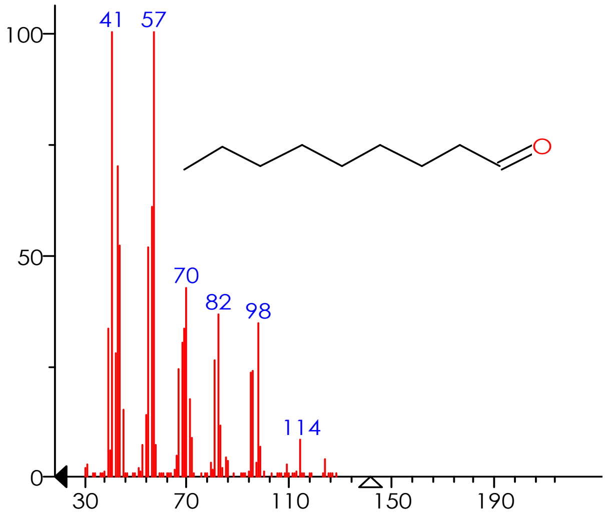

Fig. S2-continued.

61

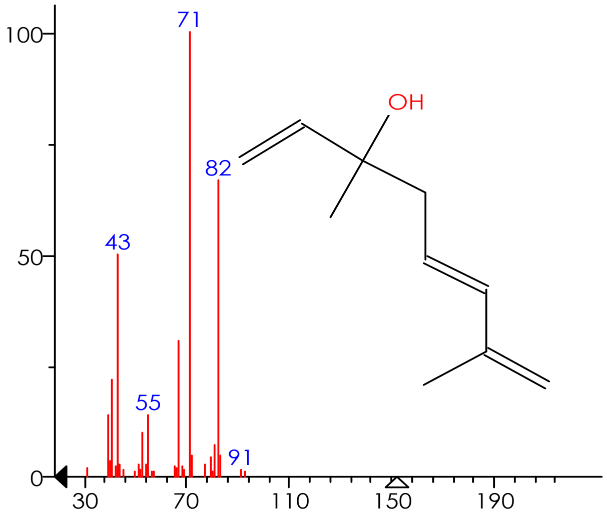

63

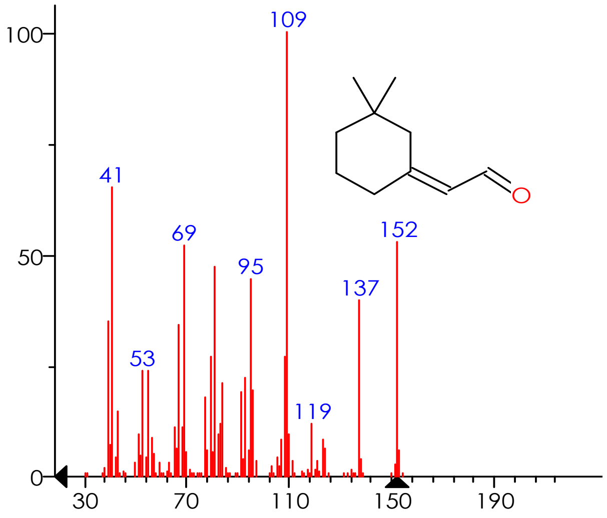

64

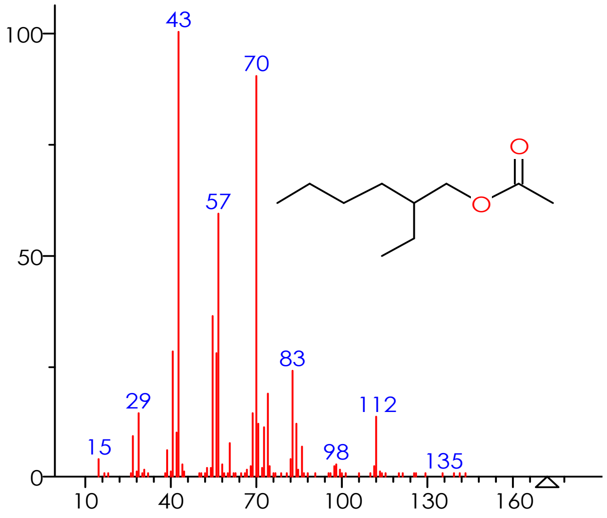

65

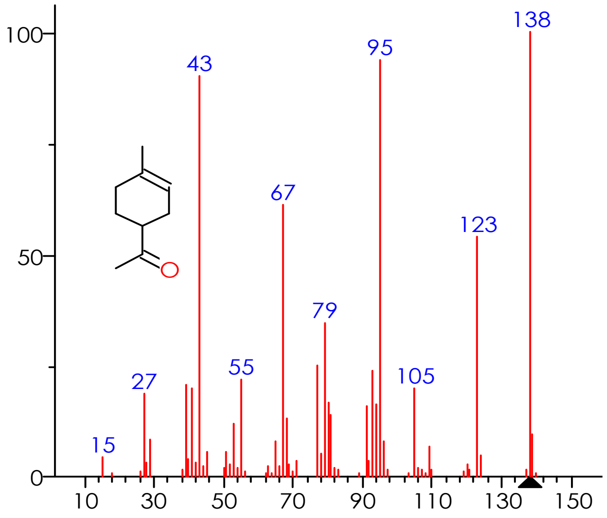

66*

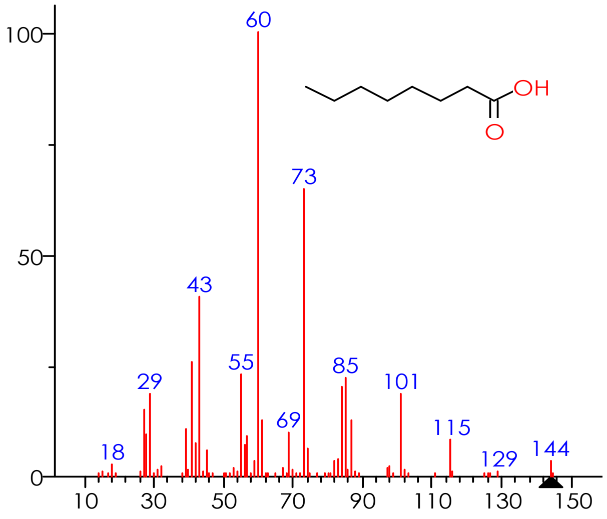

67

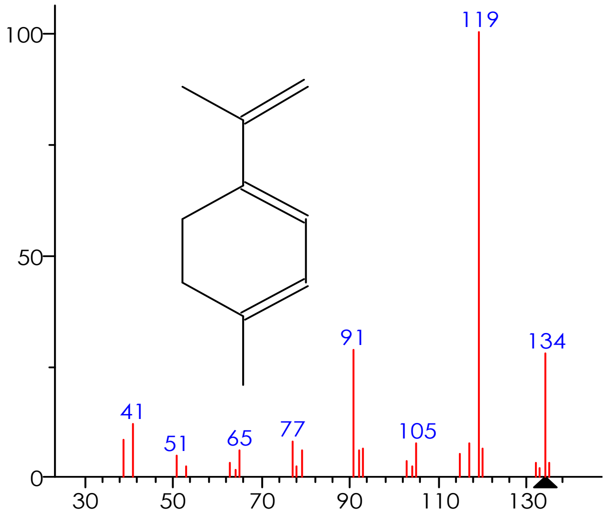

Fig. S2-continued.

70*

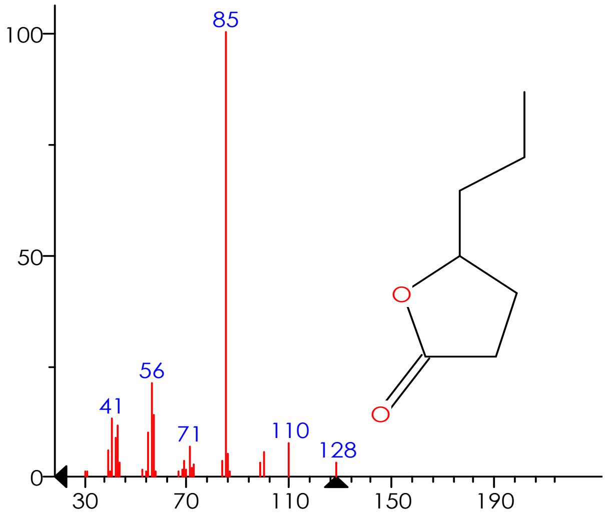

71*

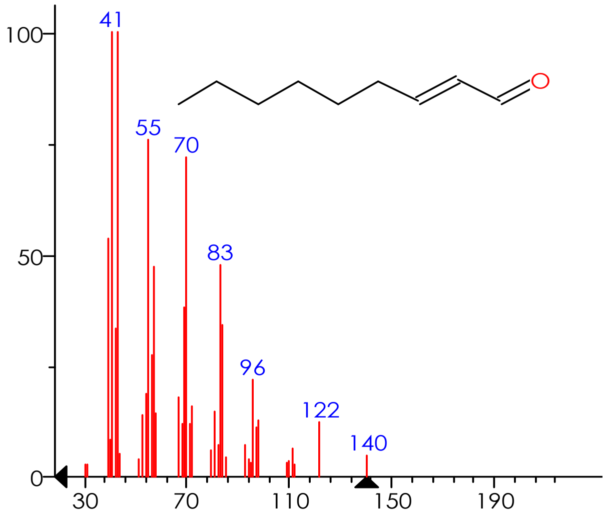

72

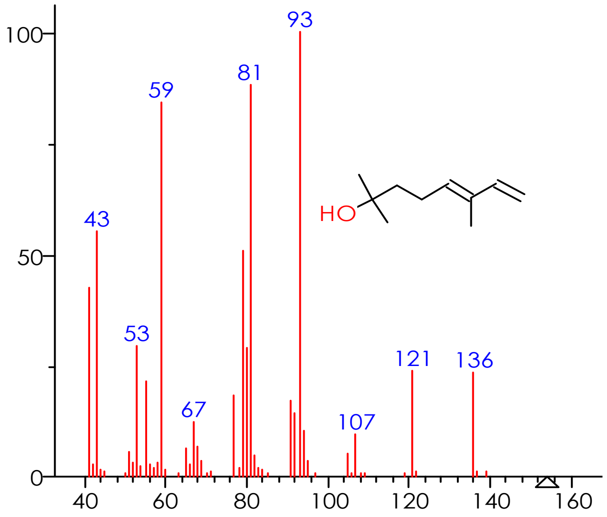

73*

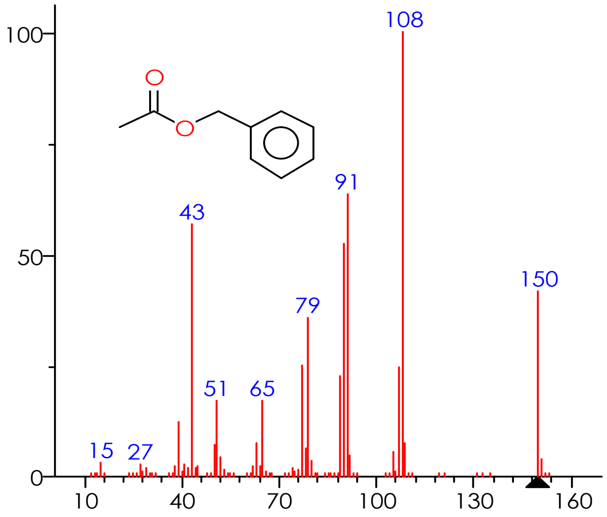

74

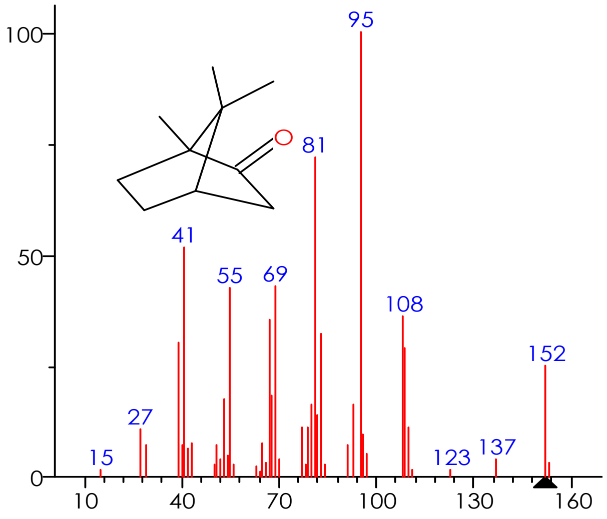

77*

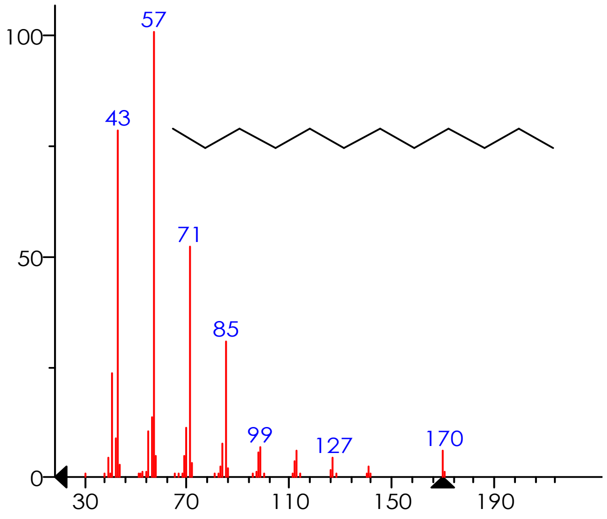

Fig. S2-continued.

78

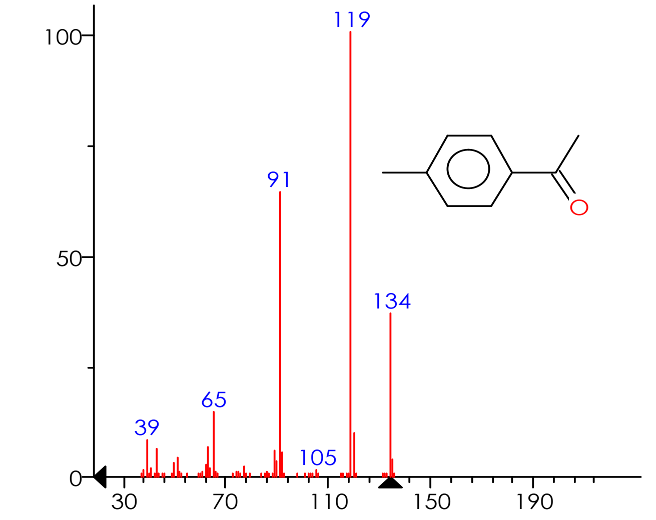

79*

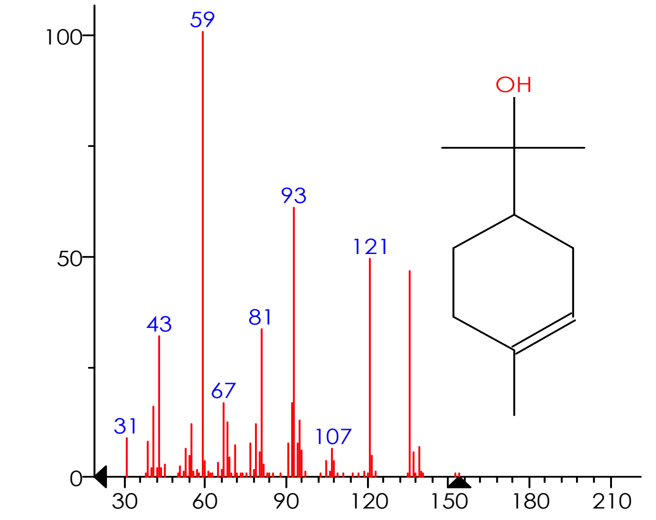

81

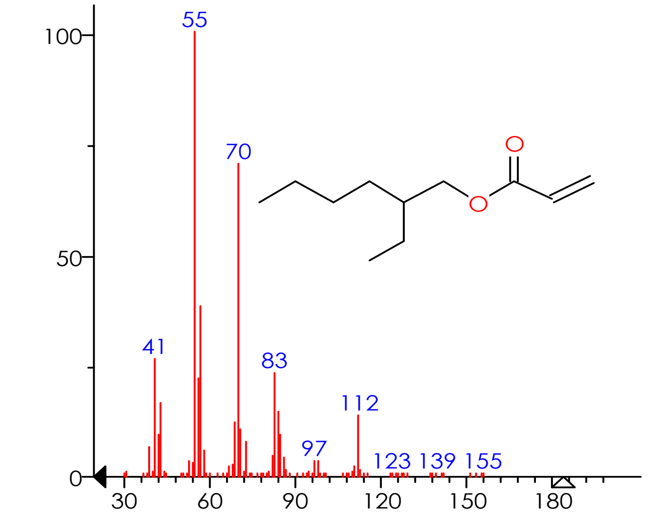

82

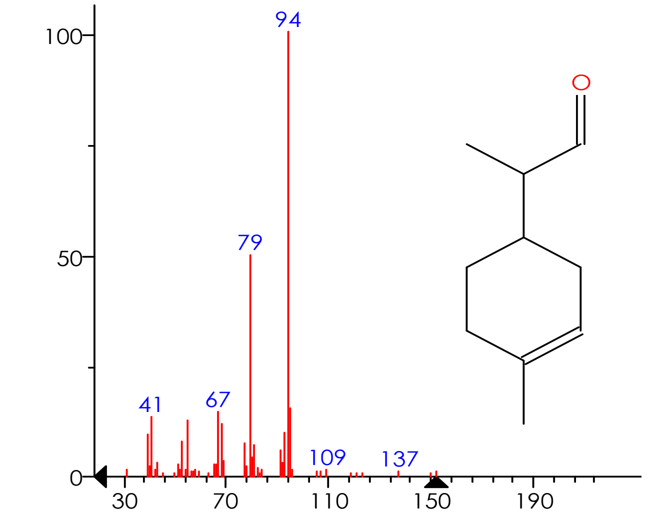

83

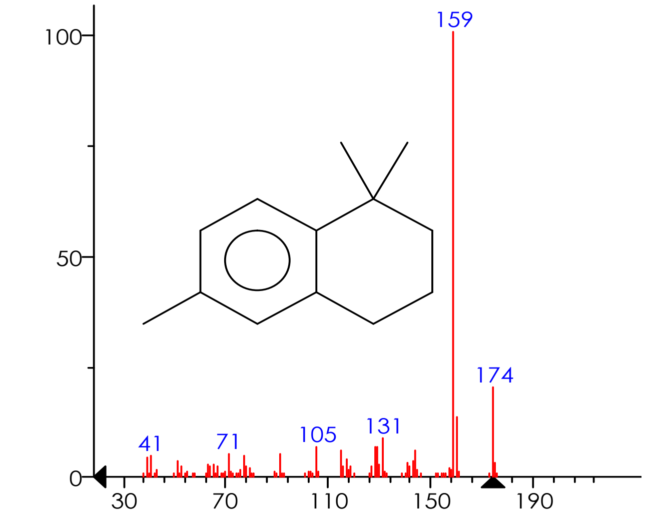

84*

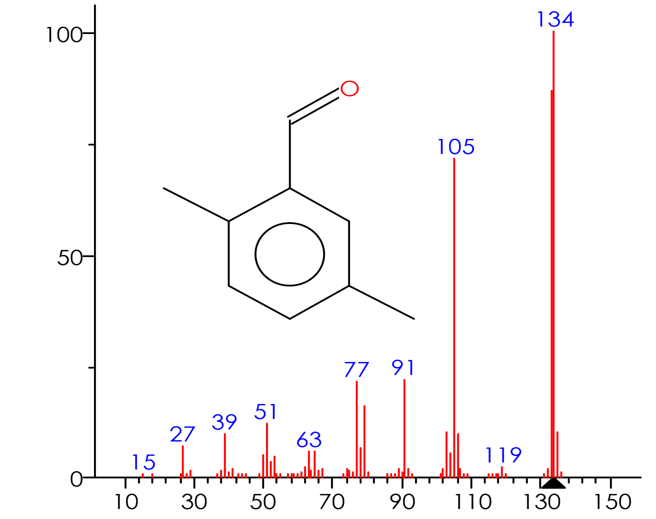

Fig. S2-continued.

87*

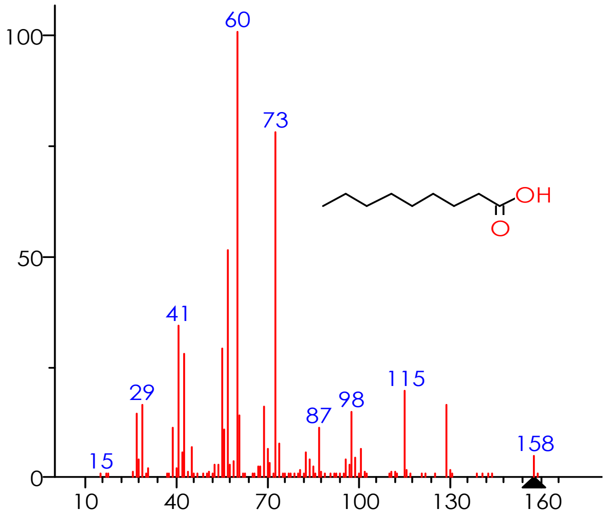

88

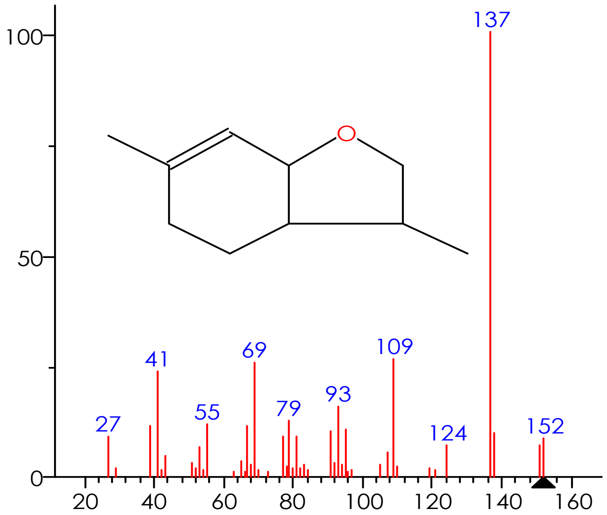

89*

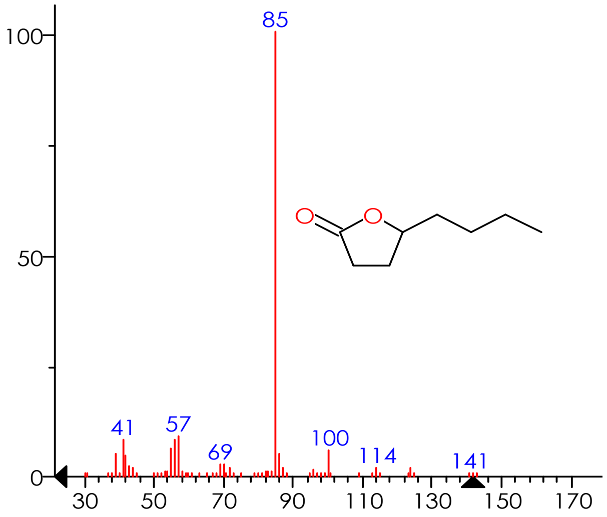

90*

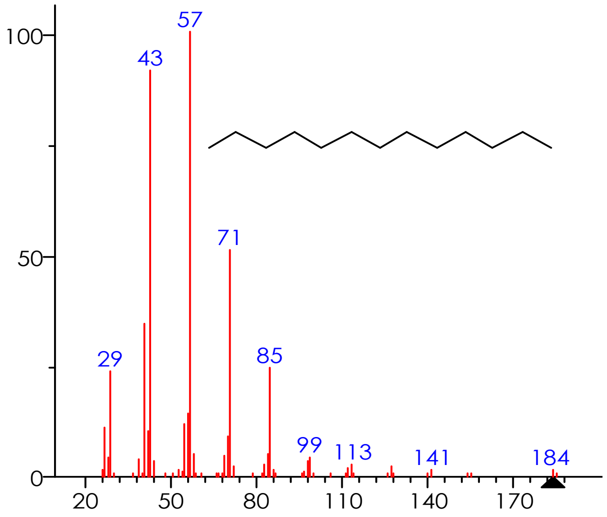

91

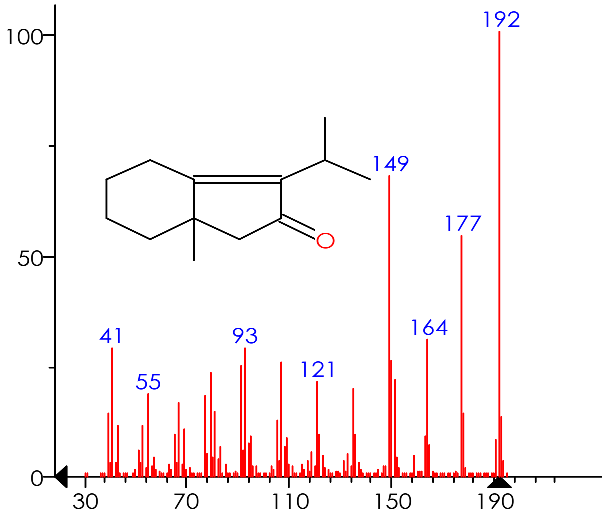

93*

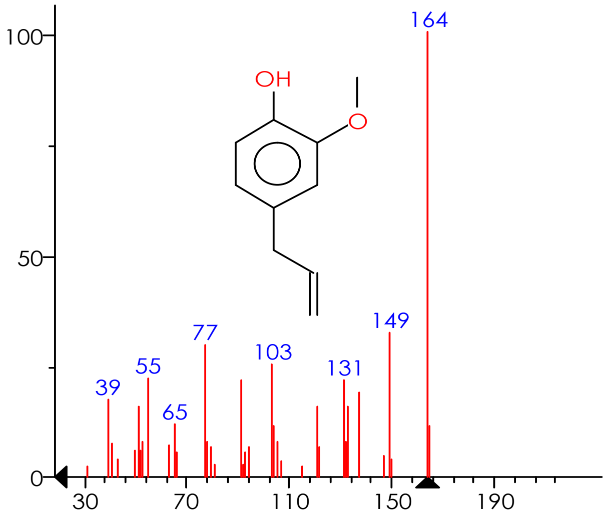

Fig. S2-continued.

95*

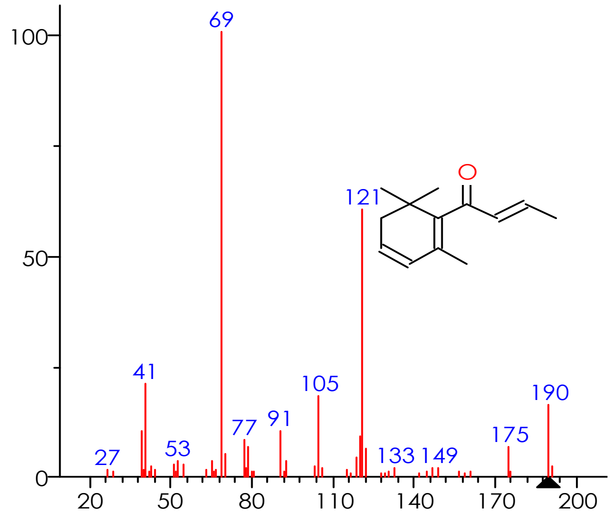

96

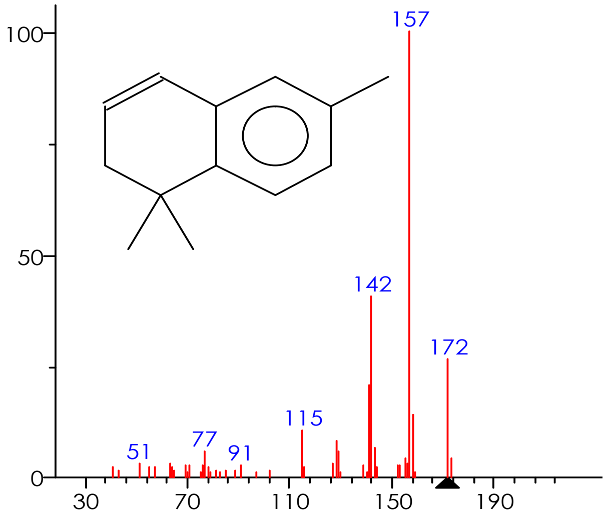

97

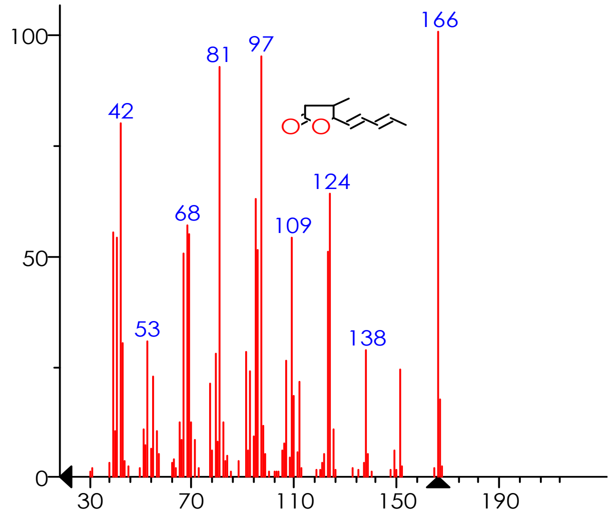

99*

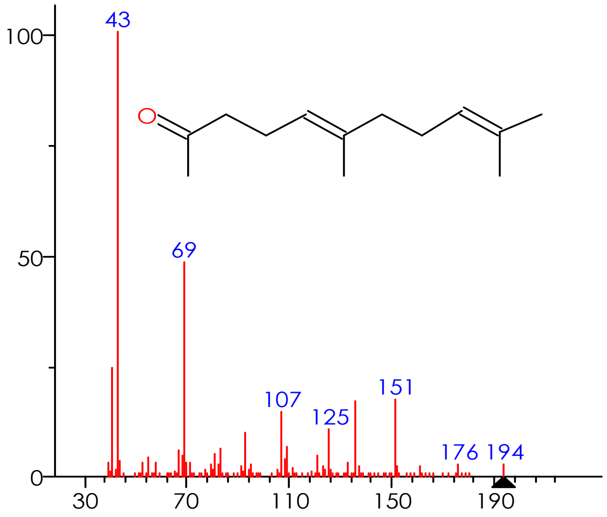

100*

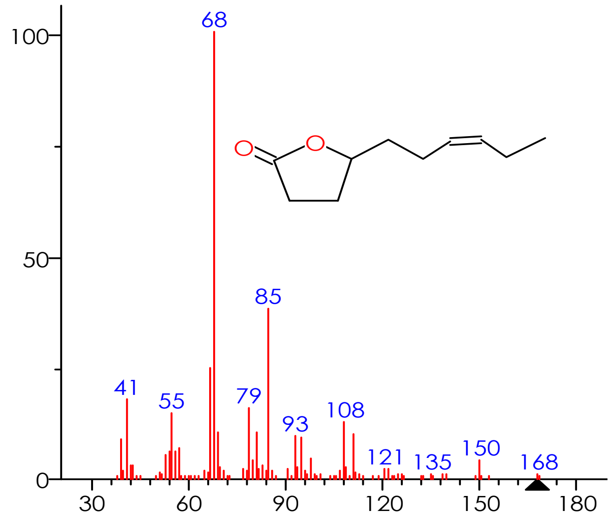

102*

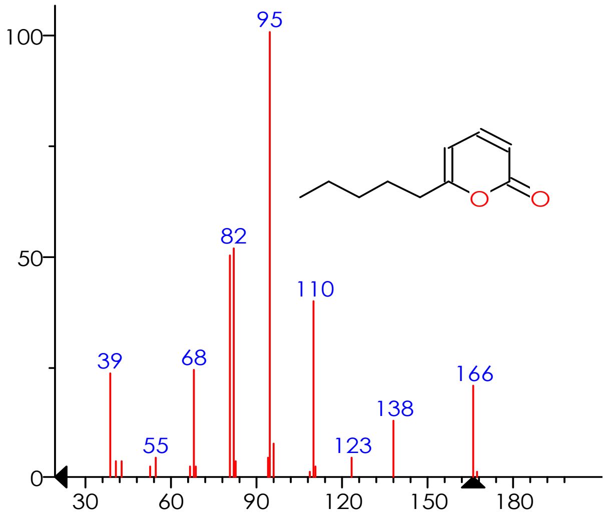

Fig. S2-continued.

103*

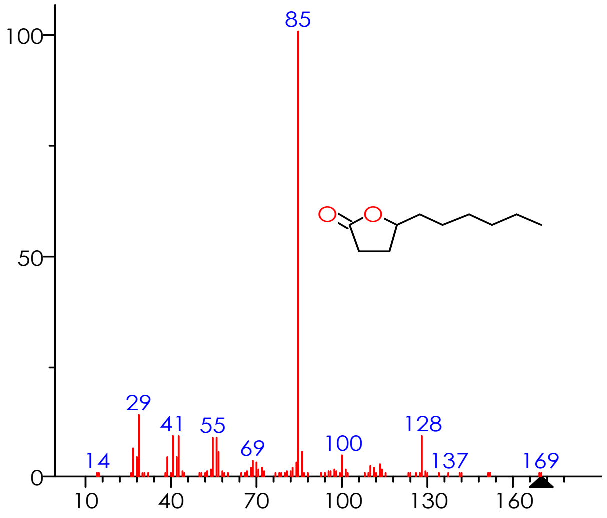

105*

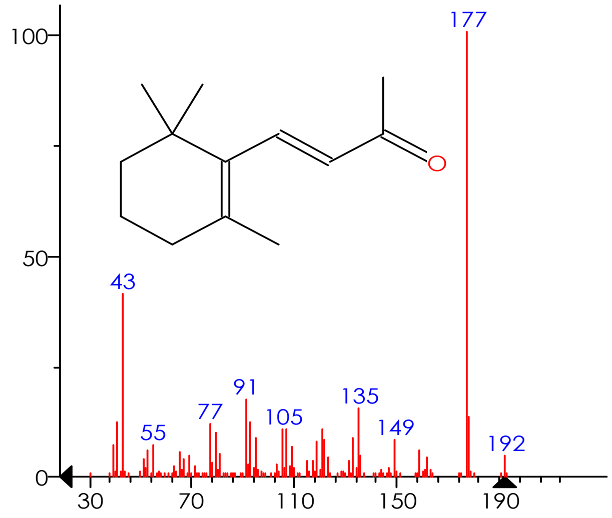

106*

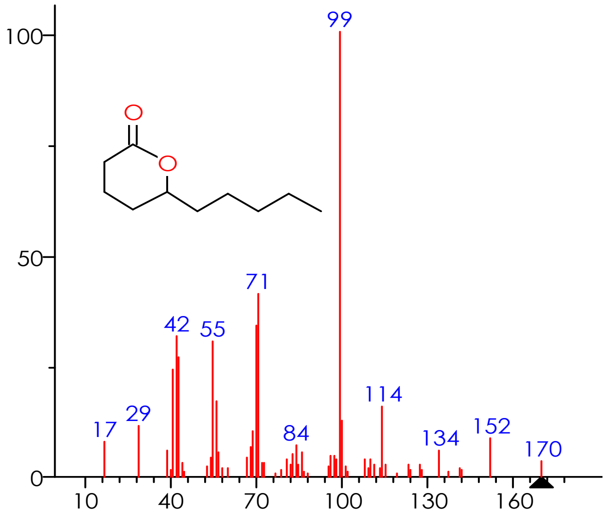

107

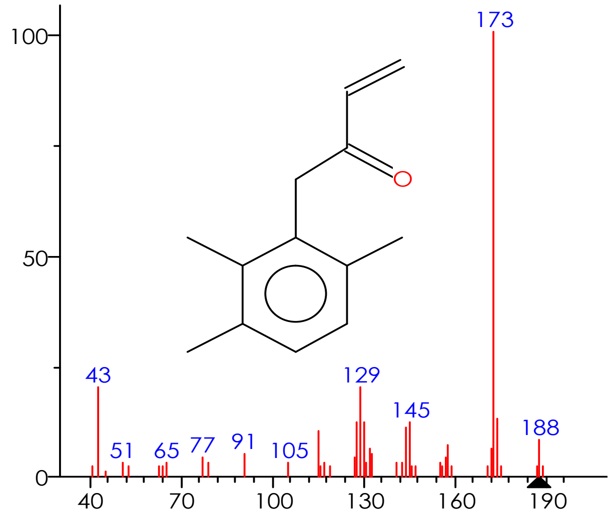

109

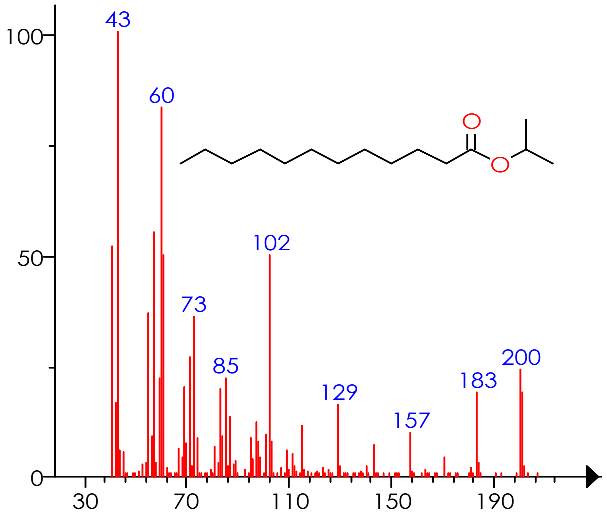

110*

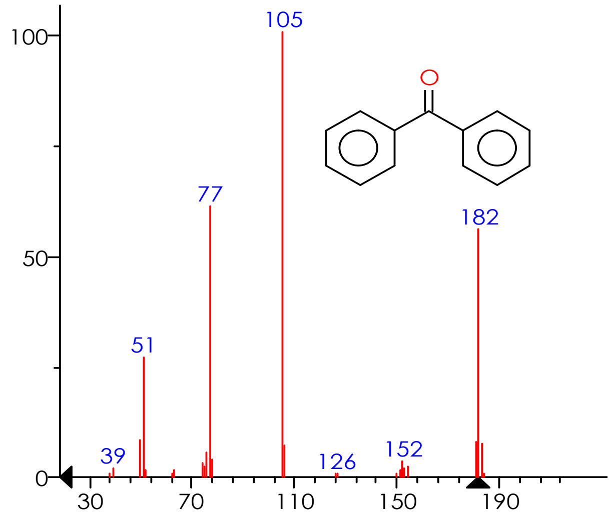

Fig. S2-continued.
