# Supplementary material for: A Non-Targeted Approach Unravels the Volatile Network in Peach Fruit
Source: PLoS One. 2012 Jun 22;7(6):e38992. doi: 10.1371/journal.pone.0038992 (PMC3382205; doi:10.1371/journal.pone.0038992)
Supplement: Table S1 — Volatile organic compounds detected in the sample set. For each volatile, the retention time (RT) in min, the cluster (Cl) that it belongs to according to Fig. 1, the specific ion (Ion, m/z) used for quantification, the forward (F) and reverse (R) matches against the Nist library (with the exception of γ-Jasmolactone, where the F and R matches against its authentic standard are shown) and the CAS number are indicated. *compound identified by comparing its retention time with an authentic standard are highlighted in bold. aThe full name of compound N°91 is: 1H-2-Indenone,2,4,5,6,7,7α-hexahydro-3-(1-methylethyl)-7α-methyl. na, not available. (PDF) [file pone.0038992.s003.pdf]

Table S1: Volatile organic compounds detected in the sample set.

Nº	RT (min)	CI	VOCs*	Ion	Nist Match (F)	Nist Match (R)	CAS Nº
1	5.659	6	**Ethanol**	45	842	856	64-17-5
2	7.785	5	Pentane, 2-methyl-	71	880	905	107-83-5
3	8.201	5	Pentane, 3-methyl-	57	877	887	96-14-0
4	9.051	8	3-Buten-2-ol, 2-methyl-	71	834	883	115-18-4
5	9.108	8	**Ethyl Acetate**	43	871	876	141-78-6
6	9.410	4	Pentane, 2,2-dimethyl-	57	806	905	590-35-2
7	9.731	4	Cyclopentane, methyl-	56	848	876	96-37-7
8	11.545	4	**1-Penten-3-one**	55	816	866	1629-58-9
9	12.023	3	**Pentanal**	44	932	942	110-62-3
10	12.110	4	**Furan, 2-ethyl-**	81	821	875	3208-16-0
11	14.297	1	2H-Pyran, 3,4-dihydro-6-methyl-	83	815	847	16015-11-5
12	14.798	7	**Acetic acid, 2-methylpropyl ester**	56	849	875	110-19-0
13	15.244	11	Propanoic acid, 2,2-dimethyl-	57	875	906	75-98-9
14	15.925	4	**3-Hexenal, (*Z*)-**	69	831	835	6789-80-6
15	16.028	4	**Hexanal**	72	922	922	66-25-1
16	16.413	11	Acetic acid, butyl ester	43	913	922	123-86-4
17	16.862	3	**Butanoic acid, 3-methyl-**	60	862	875	503-74-2
18	17.216	6	**Butanoic acid, 2-methyl-**	74	807	848	116-53-0
19	17.828	4	Ni_01	83	<800	<800	na
20	18.211	4	**2-Hexenal**	83	927	928	6728-26-3
21	18.315	11	**Pentanoic acid**	60	801	890	109-52-4
22	21.350	9	Ni_02	69	<800	<800	na
23	22.134	4	**2-Heptenal, (Z)-**	83	915	943	57266-86-1
24	22.137	10	**Hexanoic acid**	60	833	866	142-62-1
25	22.373	9	Ni_03	71	<800	<800	na
26	22.753	6	**Benzaldehyde**	77	832	869	100-52-7
27	22.864	1	2H-Pyran, 2-ethenyltetrahydro-2,6,6-trimethyl-	139	812	833	7392-19-0
28	22.952	2	1,4-Cyclohexadiene, 3-ethenyl-1,2-dimethyl-	119	840	863	62338-57-2
29	23.031	4	**5-Hepten-2-one, 6-methyl-**	108	847	893	110-93-0
30	23.279	1	**β-Myrcene**	93	820	842	123-35-3
31	23.350	3	**Furan, 2-pentyl-**	81	805	852	3777-69-3
32	23.365	9	Orthoformic acid, triisobutyl ester	57	803	905	16754-49-7
33	23.614	7	**3-Hexen-1-ol, acetate, (*Z*)-**	67	908	908	3681-71-8
34	23.731	2	Benzene, 1-ethyl-3,5-dimethyl-	119	802	835	934-74-7
35	23.783	5	**Octanal**	84	860	880	124-13-0
36	23.837	7	**Acetic acid, hexyl ester**	69	853	895	142-92-7
37	23.906	7	**2-Hexen-1-ol, acetate, (*E*)-**	67	805	831	2497-18-9
38	23.965	9	Ni_04	89	<800	<800	na
39	24.210	4	**2,4-Heptadienal, (*E*,*E*)-**	81	889	892	881395
40	24.424	1	**3-Carene**	93	819	854	13466-78-9
41	24.534	2	Naphthalene, 1,2,3,5,8,8α-hexahydro-	91	801	839	62690-65-7
42	24.631	1	1,3-Cyclohexadiene, 1-methyl-4-(1-methylethyl)-	121	832	870	99-86-5
43	24.645	3	Benzyl chloride	91	852	886	100-44-7
44	24.775	2	1-Cyclohexene-1-carboxaldehyde,2,6,6-trimethyl	109	802	823	432-25-7
45	24.893	2	Benzene, 1-methyl-2-(1-methylethyl)-	119	861	884	535-77-3
46	24.912	2	**p-Cymene**	119	803	860	99-87-6
47	24.994	5	3,5-Octadien-2-ol	111	834	857	69668-82-2
48	25.112	1	**δ-Limonene**	68	847	856	138-86-3
49	25.504	10	**Heptanoic acid**	60	852	881	111-14-8
50	25.632	5	**Benzeneacetaldehyde**	91	829	884	122-78-1
51	25.774	7	**γ-Hexalactone**	85	843	888	695-06-7
52	25.779	6	Ni_05	70	<800	<800	na
53	26.187	5	2-Cyclohexen-1-one, 3,5,5-trimethyl-	82	808	850	78-59-1
54	26.477	2	**cis-Linaloloxide**	59	840	916	na
55	26.998	3	Ni_06	95	<800	<800	na
56	27.047	10	**Hexanoic acid, 2-ethyl-**	73	838	851	149-57-5
57	27.049	1	**Terpinolene**	121	801	854	586-62-9
58	27.175	2	Benzene, 4-ethenyl-1,2-dimethyl-	132	865	924	27831-13-6
59	27.184	1	**Linalool**	93	830	847	78-70-6
60	27.295	5	**Nonanal**	57	817	851	124-19-6
61	27.319	2	1,5,7-Octatrien-3-ol, 3,7-dimethyl-	71	804	868	29957-43-5

62	27.491	2	Ni_07	67	<800	<800	na
63	27.994	11	Acetaldehyde, (3,3-dimethylcyclohexylidene)-,(E)	108	801	805	26532-25-1
64	28.464	9	Acetic acid, 2-ethylhexyl ester	70	822	877	103-09-3
65	28.610	2	4-Acetyl-1-methylcyclohexene	138	905	917	70286-20-3
66	28.795	10	**Octanoic Acid**	73	844	908	124-07-2
67	28.812	2	1,3,8-p-Menthatriene	119	838	886	21195-59-5
68	29.005	2	Ni_08	84	<800	<800	na
69	29.012	2	Ni_09	85	<800	<800	na
70	29.133	7	**γ-Heptalatone**	85	805	884	105-21-5
71	29.220	5	**2-Nonenal**	83	801	876	2463-53-8
72	29.333	1	5,7-Octadien-2-ol, 2,6-dimethyl-	93	872	882	5986-38-9
73	29.383	11	**Acetic acid, phenylmethyl ester**	108	804	905	140-11-4
74	29.553	11	Bicyclo[2.2.1]heptan-2-one, 1,7,7-trimethyl-, (1*S*)-	95	913	933	464-48-2
75	29.556	11	Ni_10	112	<800	<800	na
76	30.245	1	Ni_11	69	<800	<800	na
77	30.276	5	**Dodecane**	57	838	886	112-40-3
78	30.498	2	Ethanone, 1-(4-methylphenyl)-	119	832	851	122-00-9
79	30.753	1	**α-Terpinol**	121	815	827	98-55-5
80	31.025	4	Ni_12	88	<800	<800	na
81	31.067	9	2-Propenoic acid, 2-ethylhexyl ester	70	894	906	103-11-7
82	31.516	11	3-Cyclohexene-1-acetaldehyde, α,4-dimethyl-	94	815	837	29548-14-9
83	31.516	8	Naphthalene, 1,2,3,4-tetrahydro-1,1,6-trimethyl-	159	801	845	475-03-6
84	31.584	9	**Benzaldehyde, 2,5-dimethyl-**	133	833	844	5779-94-2
85	31.620	4	Ni_13	137	<800	<800	na
86	31.729	9	Ni_14	140	<800	<800	na
87	31.917	10	**Nonanoic acid**	73	889	895	112-05-0
88	32.097	2	3,6-Dimethyl-2,3,3α,4,5,7α-hexahydrobenzofuran	137	904	934	70786-44-6
89	32.451	7	**γ-Octalactone**	85	920	923	104-50-7
90	33.313	5	**Tridecane**	57	871	891	629-50-5
91	33.677	6	1H-2-Indenone,2,4,5,6,7,77α-hexahydro .....[a]	192	806	806	na
92	34.313	6	Ni_15	138	<800	<800	na
93	35.340	3	**Eugenol**	164	885	952	97-53-0
94	35.378	4	Ni_16	174	na	na	na
95	36.206	8	**β-Damascenone**	69	846	904	23726-93-4
96	36.934	8	Naphthalene, 1,2-dihydro-1,1,6-trimethyl-	157	805	889	30364-38-6
97	37.000	7	4-Methyl-5-penta-1,3-dienyltetrahydrofuran-2-one	68	823	835	185211
98	37.350	3	Ni_17	173	<800	<800	na
99	37.621	6	**Geranyl acetone**	69	803	816	3796-70-1
100	38.071	7	**γ-Jasmolactone**	68	948	948	93787-95-2
101	38.165	9	Ni_18	165	<800	<800	na
102	38.314	7	**2H-Pyran-2-one, 6-pentyl-**	95	857	906	27593-23-3
103	38.427	7	**γ-Decalactone**	85	924	924	706-14-9
104	38.486	9	Ni_19	177	<800	<800	na
105	38.883	4	**β-Ionone**	177	844	851	14901-07-6
106	39.237	7	**δ-Decalactone**	99	855	902	705-86-2
107	39.736	3	3-Buten-2-one, 1-(2,3,6-trimethylphenyl)-	173	825	874	54789-45-6
108	39.882	4	Ni_20	124	<800	<800	na
109	41.883	9	Dodecanoic acid, 1-methylethyl ester	102	805	807	10233-13-3
110	43.170	11	**Benzophenone**	182	806	917	119-61-9

For each volatile, the retention time (RT) in min, the cluster (Cl) that it belongs to according to Fig. 1, the specific ion (Ion, m/z) used for quantification, the forward (F) and reverse (R) matches against the Nist library (with the exception of γ-Jasmolactone, where the F and R matches against its authentic standard are shown) and the CAS number are indicated. *compound identified by comparing its retention time with an authentic standard are highlighted in bold. [a]The full name of compound Nº91 is: 1H-2-Indenone,2,4,5,6,7,7α-hexahydro-3-(1-methylethyl)-7α-methyl. na, not available.
